# Work ability trajectories and sick leave in individuals with post COVID-19 condition: 3-year follow-up of a population-based cohort

**DOI:** 10.1016/j.lanepe.2025.101536

**Published:** 2025-11-20

**Authors:** Tala Ballouz, Philipp Kerksieck, Sarah R. Haile, Holger Dressel, Oliver Hämmig, Georg F. Bauer, Jan S. Fehr, Milo A. Puhan, Dominik Menges

**Affiliations:** Epidemiology, Biostatistics and Prevention Institute (EBPI), University of Zurich (UZH), Hirschengraben 84, CH-8001, Zurich, Switzerland

**Keywords:** COVID-19, SARS-CoV-2, Post COVID-19 condition, Long covid, Occupation, Sick leave, Work ability, Cohort, Observational study

## Abstract

**Background:**

Data on the longer-term impact of post COVID-19 condition (PCC) on work-related functioning is limited, despite evidence on the persistence of PCC for years after infection. This study aimed to describe changes in work ability and sick leave associated with PCC up to three years post-infection.

**Methods:**

We used data from 667 working-age individuals within a prospective population-based cohort following individuals infected with SARS-CoV-2 between August 2020 and January 2021. PCC was determined at 12 months and work ability was assessed biannually. The impact of SARS-CoV-2 on participants’ physical and mental work performance and COVID-19 related sick leave were assessed at three years.

**Findings:**

Participants with protracted COVID-19 related symptoms at 12 months after infection reported persistently lower work ability scores than those without symptoms, with no evidence of a difference in change over time (−0.12 points per year, 95% CI −0.29 to 0.07). Compared to recovered individuals, work ability scores among those with moderate health impairment improved by +0.72 points per year (95% CI −0.04 to 1.46), while trends were similar among those with mild or severe impairment. A higher proportion of participants with PCC reported worsening in physical and mental performance at work than those without PCC. Among those with PCC, 11.5% (9/78) reported taking COVID-19 related sick leave for one month or more, in contrast to 4.0% (13/327) among those without PCC.

**Interpretation:**

The study highlights the prolonged impact of PCC on work-related functioning and underscores the need for targeted occupational, clinical and social measures for those affected.

**Funding:**

10.13039/501100002329Federal Office of Public Health, Department of Health of the Canton of Zurich, 10.13039/501100023510University of Zurich Foundation, Switzerland; Horizon Europe.

**Research in context**

**Evidence before this study**

We searched PubMed for all articles evaluating work ability and COVID-19 related sick leave among individuals with post COVID-19 condition (PCC), indexed up to 28 July 2025 with no language or time restrictions. The search string '(*“post covid-19” or “long covid” or “pasc” or “post-acute sequelae of SARS-CoV-2 infection”) and (“work ability” or (“sick leave” or “absenteeism”))*' was used. Of 116 articles, 23 were eligible. We found no observational studies evaluating longitudinal trajectories of work ability among individuals with PCC, or studies evaluating work ability up to three years after infection. Fifteen studies assessed sick leave and eight evaluated more broadly absenteeism related to COVID-19 or PCC. Most studies focused on healthcare workers, individuals hospitalised for COVID-19, or patients with PCC recruited through post COVID-19 clinics. The reported proportion of individuals requiring sick leave varied significantly, from 1.4% to 92.3%, related to differences in underlying populations, definitions, and assessment timepoints. Factors associated with sick leave included female sex, older age, obesity and having pre-existing comorbidities such as chronic pulmonary diseases.

**Added value of this study**

To our knowledge, this is the first study to examine work ability over a three-year course in a population-based cohort of working-age individuals with and without PCC. We found persistently lower work ability scores among participants with protracted COVID-19 related symptoms at 12 months post-infection compared to those without symptoms, with no relevant change up to three years. Participants with moderate health impairment showed an improvement in work ability scores (0.72 points per year) relative to those fully recovered, whereas no meaningful changes were seen among individuals with mild or severe health impairment. COVID-19 related sick leave of one month or longer was reported by 4% of individuals without protracted symptoms compared with 11.5% among those with symptoms.

**Implications of all the available evidence**

Individuals affected by PCC consistently reported lower work ability than those without PCC, with no relevant change over time. While we observed some improvement among those with moderate health impairment, those with severe impairment continued to experience persistently low work ability, indicating a subgroup of people at risk of prolonged occupational limitations. These findings underscore the long-term impact of PCC on work-related outcomes and highlight the need for timely interventions including tailored rehabilitation services, workplace accommodations, and adequate financial support. Such measures are essential to support the recovery and well-being of affected individuals and to mitigate long-term socioeconomic consequences.

## Introduction

A significant proportion of working individuals affected by post COVID-19 condition (PCC)—or Long Covid—experience substantial and prolonged limitations in their work capacity.^1^ Studies have indicated that individuals affected with PCC were more likely to reduce their work hours,^2^, ^3^, ^4^, ^5^ shift job roles,^6^ or even leave employment entirely^2^, ^3^, ^4^ within the first year post-infection. PCC-associated reductions in productivity, increased absenteeism, presenteeism, and early workforce exit have been estimated to result in billions of dollars in annual economic losses,^5^^,^^7^, ^8^, ^9^ although these estimates may also reflect broader pandemic disruptions^10^ and post-pandemic crises.^11^

Long-term studies have demonstrated that, while most individuals eventually recover from PCC, up to 15% still report protracted symptoms for at least two years after infection.^12^, ^13^, ^14^ Much of this existing literature has focused on describing longer-term symptom patterns and clinical trajectories related to PCC. However, few evaluated longer-term occupational implications.^1^ Consequently, the economic and societal impact may be underestimated if work impairments worsen or persist longer than projected, or overestimated if they resolve quickly and individuals are able to fully reintegrate into work sooner than anticipated.

Longitudinal research is therefore essential to clarify whether PCC-associated impairments in work ability improve, persist, or even worsen over time. Such studies are critical for capturing the full scope of the burden of PCC and for informing public health strategies to mitigate long-term functional losses, including early retirements and prolonged occupational disability. To address this, our study aimed to describe trajectories in work ability and impaired performance at work associated with PCC up to three years post-infection in a working-age population within a prospective population-based cohort.

## Methods

### Study design and participants

We used data from a prospective, population-based, observational cohort of individuals with diagnosed SARS-CoV-2 infection from the Canton of Zurich, Switzerland (Zurich SARS-CoV-2 Cohort; ISRCTN14990068^15^). Based on mandatory reporting of all SARS-CoV-2 infections to the Department of Health of the Canton of Zurich, we prospectively and daily invited an age-stratified (18–39 years, 40–64 years, ≥65 years), random sample of eligible individuals diagnosed between 06 August 2020 and 19 January 2021 for study participation. Eligibility criteria were being 18 years or older, able to follow study procedures, residing in Zurich, and having sufficient knowledge of the German language. All participants were enrolled upon or shortly after diagnosis, infected with the ancestral SARS-CoV-2 variant, and unvaccinated at time of infection. As in our previous work,^6^ we included individuals of working age (18–64 years old; retirement age is 65 years in Switzerland) who did not report being retired at enrolment and who did not report a reinfection event. The study was approved by the ethics committee of the Canton of Zurich (BASEC-Nr. 2020-01739) and we obtained written or electronic consent from all participants.

### Data sources

We collected data through electronic questionnaires. The baseline questionnaire, administered immediately after enrolment, included questions on sociodemographics, the acute primary infection (i.e., symptoms, severity), pre-existing comorbidities (hypertension, diabetes mellitus, cardiovascular disease, respiratory disease, chronic renal disease, current or past malignancy, and immune suppression), and self-rated health status prior to infection. Participants’ health trajectories (e.g., new infections and symptoms) were assessed at regular six-month intervals throughout follow-up. Starting at 12 months post-infection and every six months thereafter, we additionally collected data on work ability and receipt of health services (specifically psychological support and counseling, physiotherapy, occupational therapy, pulmonary/respiratory rehabilitation, general physical rehabilitation, and other services*)* related to PCC. At the 36-month follow-up, we further asked participants whether their physical or mental work performance has changed compared to pre-infection levels, whether they attributed these changes to the SARS-CoV-2 infection, and about the duration of any COVID-19 related sick leave. Response rates from 12 months to 36 months ranged between 82.8% and 62.4% (Supplementary Table S1). Participants initially consented to a one-year participation period and were asked to consent for further biannual assessments following each of the 12-month and 24-month questionnaires.

### Outcome measurement

We assessed self-perceived work ability using selected items from the Work Ability Index, an established instrument for assessing work ability.^16^, ^17^, ^18^ Our primary outcome was the work ability score (range 0–10, 10 being best ability and 0 no ability to work) over time. Secondary outcomes included categorised work ability (using score cut-offs: poor ≤6, moderate 7–8, and excellent ≥9^19^), work ability related to physical and mental demands (5-point Likert scale), and estimated future work ability in two years (3-point Likert scale) over time, as well as self-reported changes in physical or mental performance at work at 36 months compared to pre-infection levels (5-point Likert scale). Additionally, we assessed self-reported COVID-19 related sick leave and its duration.

We defined PCC at 12 months of follow-up using two distinct measures for better comparability with the existing heterogenous literature. The first measure, *self-reported COVID-19 related symptoms*, was based on the presence of any of 23 symptoms commonly associated with PCC and reported by participants to be related to COVID-19 at 12 months. The second measure, *(non-)recovery and health impairment*, combined participants’ self-reported recovery status (fully recovered and symptom-free vs non-recovered) and their health status at 12 months (assessed using EuroQol visual analogue scale (EQ-VAS)). Non-recovered participants were categorised into mild (EQ-VAS >70), moderate (EQ-VAS 51–70) and severe health impairment (EQ-VAS ≤50). Additionally, we evaluated the following PCC-related symptom clusters often referred to in the literature: fatigue/physical exertion, cardiorespiratory (defined as dyspnoea, palpitation, or chest pain), or neurocognitive symptoms (defined as concentration, memory, or sleeping problems). Additional details on question wording and categorisation of outcomes are provided in the Supplementary Methods.

### Statistical analysis

We used descriptive analyses for participant characteristics and all outcomes. Results are presented as means with standard deviations (SD) and medians with interquartile ranges (IQR) for continuous variables and proportions with Wilson's 95% confidence intervals (CI) for categorical variables.

For work ability score trajectories (as a continuous outcome) over time, we used robust linear mixed effects models to test the association with presence of PCC (using symptom based and non-recovery definitions and symptom clusters) at 12 months. In these models, time (in years), PCC status and their interaction were included as fixed-effect predictors. The models were further adjusted for age (as continuous variable), sex, education, comorbidity count (dichotomized), presence of psychiatric history, hospitalisation status during acute infection, and baseline health status (EQ-VAS at baseline), using a random intercept to account for within-person correlation. A robust linear mixed model was chosen due to observed deviations from normality and the presence of outliers. Such models reduce the effect of outliers but still maintain ease of interpretability similar to standard linear models.^20^ We used estimated marginal means derived from the main robust mixed models to estimate adjusted average scores for visualising changes in work ability scores over time. For all models, CIs were calculated using stratified non-parametric bootstrapping with 1000 iterations.

We further applied stabilised inverse probability of censoring weighting (IPCW) to all estimates from primary and secondary outcomes to account for potential emigrative selection bias introduced by missing data due to losses to follow-up. IPCW weights at each follow-up timepoint were derived using logistic regression models predicting the probability of non-missingness at that time point. We selected covariables a priori and considered the following to be related to missingness and work ability: age, sex, body mass index, smoking status, education level, monthly income, comorbidity count (as continuous variable), and presence of PCC (using corresponding definitions).

To assess the robustness of our findings, we conducted several sensitivity analyses: for descriptives, we applied (non-weighted) available case analysis and complete case analysis; for statistical models, we performed standard linear mixed models, an analysis restricted to participants who were employed or self-employed at baseline, an analysis using multiple imputation, and an analysis using multiple imputation with delta-adjustment to explore plausible scenarios of non-random missingness (Supplementary Methods).

We further conducted subgroup analyses to examine whether the association between symptom-based PCC status and work ability differed by prespecified participant subgroups. Interaction terms with sex, age group, comorbidity count, and presence of psychiatric history were included to evaluate between-group differences at baseline and differences in trends over time. Subgroup analyses were not possible for the non-recovery definition of PCC due to sparse data in some subgroups.

To identify risk factors associated with ever having COVID-19 related sick leave, we conducted multivariable logistic regression analyses. Models included age, sex, comorbidity count (dichotomized), baseline health status, history of psychiatric diagnosis, education level, hospitalisation during acute infection, symptom count during acute infection, and presence of COVID-19 related symptoms at 12 months, selected a priori as covariates. As a sensitivity analysis, we repeated the analysis restricted to participants who were employed or self-employed at baseline.

All analyses were performed in R (version 4.5.1).

### Role of the funding source

The funders of the study had no role in study design, data collection, data analysis, data interpretation, decision to publish, or writing of the report.

## Results

A total of 3185 individuals were randomly sampled and invited to participate in the Zurich SARS-CoV-2 Cohort, of which 1106 consented for participation (participation rate 34.7%; Supplementary Figure S1). 306 of the 1106 participants were not part of the working-age population, 10 were excluded due to reinfection, and 113 did not provide data at 12 months. Of 667 participants included in this study, 362 (54.3%) were female, 386 (57.9%) were aged 40–64 years, 78 (11.7%) had an asymptomatic SARS-CoV-2 infection, and 9 (1.3%) were hospitalised (Table 1). 19 participants (2.8%) reported being unemployed and 4 (0.6%) reported receiving disability insurance benefits at baseline.

**Table 1:** Characteristics of the study population.

	Overall
	(N = 667)
**Age**	
Median (IQR)	43.0 (31.0–53.0)
**Age group**	
18–39 years	281 (42.1%)
40–64 years	386 (57.9%)
**Sex**	
Female	362 (54.3%)
Male	305 (45.7%)
**Symptom count at infection**	
Asymptomatic	78 (11.7%)
1-5 symptoms	262 (39.3%)
≥6 symptoms	327 (49.0%)
**Hospitalisation at infection**	
Non-hospitalised	658 (98.7%)
Hospitalised	9 (1.3%)
With ICU stay	1 (0.1%)
**Smoking status**	
Non-smoker	411 (61.8%)
Ex-smoker	154 (23.2%)
Smoker	100 (15.0%)
*Missing*	*2 (0.3%)*
**BMI (kg/sqm)**	
Median (IQR)	23.7 (21.5–26.2)
*Missing*	*6 (0.9%)*
**Comorbidity**	
None	528 (79.2%)
1 comorbidity	113 (16.9%)
2+ comorbidities	26 (3.9%)
**History of psychiatric diagnosis**	
No	565 (86.8%)
Yes	86 (13.2%)
*Missing*	*16 (2.4%)*
**Education level**	
None or mandatory school	22 (3.3%)
Vocational training or specialised baccalaureate	247 (37.1%)
Higher technical school or college	193 (29.0%)
University	203 (30.5%)
*Missing*	*2 (0.3%)*
**Employment at infection**	
Employed or self-employed	582 (87.3%)
Student	46 (6.9%)
Housewife/family manager	10 (1.5%)
Unemployed	19 (2.8%)
Disability insurance benefits	4 (0.6%)
Other	6 (0.9%)
**Monthly household income**	
<6′000 CHF	188 (29.2%)
6′000–12′000 CHF	281 (43.6%)
>12′000 CHF	175 (27.2%)
*Missing*	*23 (3.4%)*
**Nationality**	
Swiss	559 (83.8%)
Non-Swiss	108 (16.2%)
**COVID-19 related symptoms at 12 months**	
No symptoms	547 (82.0%)
Symptoms	120 (18.0%)
**(Non-) recovery and health impairment at 12 months**	
Recovered	562 (85.8%)
Mild	72 (11.0%)
Moderate	13 (2.0%)
Severe	8 (1.2%)
*Missing*	*12 (1.8%)*

Legend: IQR, interquartile range; SD, standard deviation; CHF, Swiss Francs.

With respect to PCC, 120 (18.0%) participants reported having COVID-19 related symptoms, and 93 (14.2%) reported not having recovered at 12 months. A higher proportion of participants categorized as having PCC were aged 40–64 years, females, had ≥6 symptoms during acute infection, at least one comorbidity, or a history of psychiatric diagnosis compared to those without PCC (Supplementary Table S2).

A total of 22 participants reported receiving at least one health service related to PCC during the three-year follow-up period. Specifically, among those with COVID-19 related symptoms, 11 participants reported psychological support and counselling, 10 physiotherapy, six general physical rehabilitation, two pulmonary/respiratory rehabilitation, four occupational therapy, and 13 other (non-specified) health services.

### Work ability scores over time

Participants with COVID-19 related symptoms at 12 months reported persistently lower work ability scores over time compared to those without symptoms (Supplementary Table S3). In both groups, mean scores remained relatively stable over time, ranging from 8.86 (SD 1.54) at 12 months to 8.78 (1.46) at 36 months among participants without symptoms and 7.88 (2.21) at 12 months to 7.55 (2.32) at 36 months among participants with symptoms. Similarly, participants who reported being recovered at 12 months had consistently high work ability scores over time (8.93 (1.43) at 12 months and 8.80 (1.42) at 36 months) while those with mild health impairment showed slightly lower but also relatively stable scores over time (8.36 (1.03) at 12 months and 7.86 (1.86) at 36 months). Participants with moderate or severe health impairment had consistently lower scores than those with mild health impairment. The scores of those with moderate health impairment increased over time (4.92 (2.10) at 12 months to 6.23 (2.26) at 36 months), while those with severe health impairment had persistently low work ability scores (3.38 (2.93) at 12 months to 2.49 (3.22) at 36 months). The overall trend of scores over time was similar across sensitivity analyses, albeit with generally slightly higher work ability scores in complete case analyses (Supplementary Table S3).

**Fig. 1:**
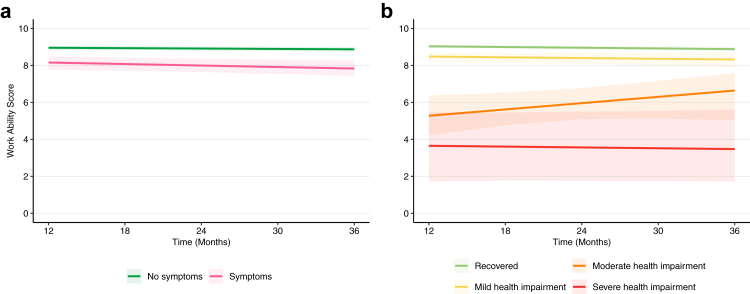
**Average work ability scores over time by presence of PCC status at 12 months (defined as COVID-19 related symptoms in panel a, and as (non)-recovery and health impairment in panel b)**. Average scores were estimated using robust linear mixed effects models adjusted for age, sex, education level, comorbidity count, presence of psychiatric history, hospitalisation status during acute infection, and baseline health status. Shaded areas represent 95% confidence intervals derived using bootstrapping.

Based on robust linear mixed effects models, we found evidence that mean work ability scores were lower at baseline by 0.68 points (95% CI −1.16 to −0.25) among those with COVID-19 related symptoms compared to those without. Meanwhile, we found no evidence for a greater or smaller change in scores over time relative to those without symptoms (−0.12 points per year, 95% CI −0.29 to 0.07) (Fig. 1A, Supplementary Table S4). In sensitivity analyses, similar results were observed when restricting to individuals who were employed or self-employed at baseline (Supplementary Table S5). Consistent with the main analysis, analyses using multiple imputation showed no evidence for a difference in change over time among participants with COVID-19 related symptoms compared to those without. Although estimates were somewhat smaller then with IPCW (multiple imputation without delta adjustment: −0.06; delta = +1: −0.05; delta = +2: −0.01; delta = −1: −0.03; delta = −2: 0.00), this indicates that the findings were overall robust to varying assumptions about missingness (Supplementary Table S4).

Compared to the recovered group, those with moderate health impairment improved by +0.72 points per year (95% CI −0.04 to 1.46), while work ability scores in those with mild (0.00 per year, −0.21 to 0.18) and severe (−0.00 per year, −0.40 to 0.33) health impairment did not show a difference in changes over time (Fig. 1B, Supplementary Table S6). In sensitivity analyses restricting to those employed at baseline and using multiple imputation and applying delta adjustment, findings were again similar to the main analysis, with some variation in the magnitude of improvement among participants with moderate health impairment across different scenarios (Supplementary Table S6).

When analysing the presence of symptom clusters, we found no evidence of a difference in the change in scores over time among participants reporting symptoms across any of the three symptom clusters (fatigue/physical exertion: −0.06 per year, −0.40 to 0.30 for; cardiorespiratory: 0.16 per year, −0.26 to 0.59; neurocognitive: −0.18 per year, −0.58 to 0.29) compared to those without respective symptoms (Supplementary Table S7).

In subgroup analyses, there was evidence for a difference in the association of COVID-19 symptoms and work ability scores by history of psychiatric diagnosis, with a larger reduction in work ability scores among individuals with a prior diagnosis compared to those without (difference = −1.72, 3.26 to −0.32). Work ability scores were also more strongly reduced among participants aged 40–65 years compared to those aged 18–39 years (−0.56, −1.44 to 0.39) and among those with ≥2 comorbidities to those with 0–1 comorbidity (−1.17, −5.01 to 1.72), although confidence intervals were wide and overlapped with 0. Across all subgroups, there was no evidence of effect modification on the difference in changes in scores over time (Supplementary Table S8, Supplementary Figure S2).

### Work ability categories, work ability related to physical and mental demands, and estimated future work ability

Most participants without COVID-19 related symptoms consistently reported excellent work ability across timepoints (69.5%, 95% CI 65.4%–73.2% at 12 months and 69.7%, 64.6%–74.4% at 36 months), and only a small percentage reported poor work ability (5%, 3.5%–7.2% at 12 months and 5%, 3.1%–7.9% at 36 months) (Supplementary Table S9). Meanwhile, a higher proportion of those with symptoms reported poor scores (16.8%, 11.2%–24.5% at 12 months and 19.3%, 12.2%–29.2% at 36 months). We observed similar patterns regarding non-recovery and health impairment, with consistently high proportions of participants (69.7%, 64.7%–74.3% at 36 months) reporting excellent work ability among those who had recovered at 12 months and high proportions reporting poor work ability among those with moderate (35.3%, 15.4%–62.1%) and severe (76.1%, 29.8%–96.0%) health impairment (Supplementary Table S9).

We found similar findings regarding work ability related to physical and mental demands and estimated future work ability in 2 years (Supplementary Tables S10–S12). Compared to those without, a relevant proportion of participants with COVID-19 related symptoms at 12 months reported very or rather bad work ability related to physical (6.7% vs 1.5%) and mental (5.9% vs 0.8%) demands or rated their future work ability as unlikely or not certain (16.2% vs 6.7%). These differences persisted up to 36 months and were similar for recovered vs non-recovered, with worse outcomes among those with moderate and severe health impairment compared to those with mild health impairment. Results were overall consistent across available case and complete case sensitivity analyses.

### Impaired physical and mental performance at work compared to pre-infection

**Fig. 2:**
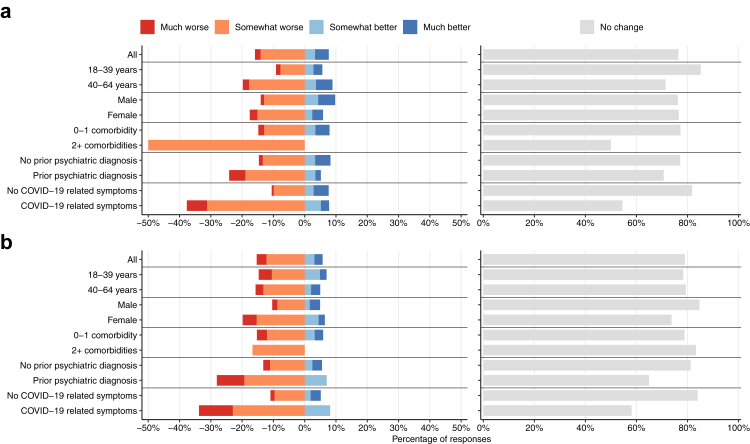
**Changes in physical (panel a) and mental (panel b) performance at work across different population subgroups**. Bars in the left-hand figures extending left of 0% represent worsening in performance, while those extending to the right of 0% indicate improvements. Gray bars in the right-hand figures represent the percentage of respondents reporting “No change”.

From the 667 included participants, 416 (62.4%) completed the 36-month follow-up questionnaire. There were no differences in the characteristics between those who completed the questionnaire and the overall sample (Supplementary Table S13). Most participants reported no change in their mental or physical performance compared to before the SARS-CoV-2 infection (Fig. 2, Supplementary Table S14). Compared to participants without COVID-related symptoms at 12 months, a higher proportion of those with symptoms reported at least somewhat worsening in their physical (37.7% vs 10.5%) and mental (33.8% vs 10.9%) performance. A higher proportion of participants aged 40–64 years and those with ≥2 comorbidities reported at least some worsening in their physical work performance compared to those aged 18–39 years (19.7% vs 9.1%) and with 0–1 comorbidities (50.0% vs 14.9%), respectively. Meanwhile, a higher proportion of women reported at least some worsening in their mental work performance compared to men (19.8% vs 10.3%).

### Sick leave due to COVID-19

A total of 284 (70.1%) participants reported ever taking sick leave due to COVID-19 or its complications including PCC (Table 2). Approximately 72% of the participants (N = 204) reported taking sick leave for two weeks or less, 22 participants (5.4%) for one month or more, and 3 participants (0.7%) taking sick leave for three months or more. Among those reporting protracted COVID-19 related symptoms, 11.5% reported taking COVID-19 related sick leave for one month or more, while this proportion was 4.0% among those without symptoms. When comparing participants reporting ever having been on sick leave with those who had not, we observed differences in sex, severity of acute infection, history of psychiatric diagnosis and presence of COVID-19 related symptoms at 12 months (Supplementary Table S15). However, we found only weak evidence of higher odds of having been on sick leave among those who had a history of psychiatric diagnosis (OR 1.81, 95% CI 0.91–3.88) and ≥6 symptoms at acute infection (OR 1.81, 95% CI 0.90–3.60) in the multivariable regression analysis (Supplementary Table S16). Results from the sensitivity analysis restricted to employed participants were overall similar, although history of psychiatric diagnosis and experiencing ≥6 symptoms at acute infection were more strongly associated with sick leave than in the main analysis (Supplementary Table S17).

**Table 2:** Proportion of participants with sick leave and duration of sick leave, among those with and without COVID-19 related symptoms.

	Overall	No COVID-19 related symptoms	COVID-19 related symptoms
	(N = 416)	(N = 332)	(N = 84)
**Ever had COVID-19 related sick leave**			
No	121 (29.9%)	104 (31.8%)	17 (21.8%)
Yes	284 (70.1%)	223 (68.2%)	61 (78.2%)
*Missing*	*11*	*5*	*6*
**Duration of COVID-19 related sick leave**			
Never (0 days)	121 (29.9%)	104 (31.8%)	17 (21.8%)
1 day-1 week	88 (21.7%)	77 (23.5%)	11 (14.1%)
1–2 weeks	116 (28.6%)	91 (27.8%)	25 (32.1%)
3–4 weeks	58 (14.3%)	42 (12.8%)	16 (20.5%)
1–3 months	19 (4.7%)	13 (4.0%)	6 (7.7%)
≥3 months	3 (0.7%)	0 (0.0%)	3 (3.8%)
*Missing*	*11*	*5*	*6*

## Discussion

We assessed work ability trajectories and sick leave up to three years post-infection in a population-based cohort of working-age individuals with confirmed SARS-CoV-2 infection. This longitudinal analysis extends on previously reported results on the association of work ability with PCC at 12 months after infection.^6^

Work ability at the population level remained relatively stable over the three-year observation period, with an expected minimal decline over time. There were significant differences persisting up to three years after infection between those without PCC and those experiencing protracted COVID-19 related symptoms or health impairment. Meanwhile, there were no relevant differences in longitudinal trends between those with COVID-19 symptoms and those without, indicating no relevant worsening or improvement over time among those with symptoms relative to those without. This is in line with findings for individuals experiencing mild health impairment, which constitute the largest subgroup of people affected by PCC in our sample: while there was a relevant difference in work ability compared to those fully recovered, we did not observe a difference in the trends over time relative to what would be expected in a general population unaffected by PCC.

In contrast, participants with moderate health impairment showed some improvement over time relative to those fully recovered. A change of 1.5–2 points in work ability scores is considered to be the minimal important difference (MID) in individuals with chronic musculoskeletal or low back pain.^21^^,^^22^ While no such MID has been established for PCC, our observed increase of around 1.5 points over two years among participants with moderate health impairment approaches these previously established thresholds, suggesting a modest, but potentially meaningful, improvement in work ability. It should be noted, however, that baseline work ability scores in the studies which established these MIDs were considerably lower (median of approximately 4) than in our cohort. Comparisons across contexts should be made cautiously, and such external MID thresholds serve only as contextual benchmarks in the absence of an established MID in PCC.

The changes observed in our study may be related to coping strategies, reduction in symptom burden, or effective interventions over the follow-up period. On the other hand, the lack of substantial increase in work ability among those with severe impairment in our study indicates a longer-lasting impact on a small subset of the workforce. This may be due to a higher symptom burden or structural barriers that hinder full recovery, such as limited return-to-work support or workplace accommodations. However, these findings should be interpreted with caution due to the small number of cases within these subgroups. Nevertheless, our study highlights the need for targeted occupational and rehabilitation interventions to support individuals experiencing persistent severe impairment which were affected by PCC early during the COVID-19 pandemic.

Several studies have described the occupational impact of PCC including a reduced work ability and delayed return-to-work,^1^, ^2^, ^3^, ^4^^,^^6^ but evidence on longer-term work-related outcomes and longitudinal trajectories is limited. A recent review found that prolonged work absences up to two years after COVID-19 were common among those with PCC.^1^ Yet, most of the identified studies were cross-sectional with a small sample size, had a follow-up time of one year or less, and had highly selected samples of individuals with PCC. To the best of our knowledge, our study is the first to report on a three-year course of work ability in a population-based cohort of individuals with and without PCC.

In our cohort, 5.4% of the total population reported ever having had sick leave related to COVID-19 for longer than one month while fewer than 1% reported extended leave durations of three months or more. Such absences from work were more frequent among individuals with protracted symptoms than without. Similar estimates of sick leave following SARS-CoV-2 have been reported by several studies.^23^, ^24^, ^25^ The observed low percentage of people requiring extended leave could be related to either a relatively low prevalence of severe health impairment, the experience of episodic or fluctuating symptoms, or the presence of effective strategies that support early reintegration into the workplace such as adjustments in work hours, workload and duties, or remote work.

However, these figures may also reflect a degree of sickness presenteeism, where individuals continue working despite their illness. This may be due to financial concerns, limited sick leave allowance or acceptance, or normalisation of working while ill.^26^ This issue is particularly relevant as we observed that around a fifth of participants—especially those with PCC, comorbidities or a history of psychiatric conditions—reported worsening in their performance at work. Hence, perceived impairments may not always lead to absences from work but can still negatively affect day-to-day productivity. This carries significant consequences, both for the employers and affected workers, including productivity losses, long-term health risks, and increased healthcare and disability costs.^27^^,^^28^ Future research should further explore presenteeism in the context of PCC and include more detailed assessments of productivity and well-being in the workplace to better capture the full occupational and economic burden of PCC.

Altogether, our findings underline the need for long-term, targeted occupational health strategies to mitigate long-term work disability following PCC and its potential consequences such as early retirement.^29^ As recovery appears to take place over years rather than weeks, future research should prioritise long term follow-up and establishing a PCC-specific MID to better evaluate occupational outcomes over time. The variation in recovery trajectories highlights the importance of moving beyond one-size-fits-all approaches through the development and provision of individualised clinical and occupational interventions.

At a societal level, reduced work ability, and consequently lower labor market participation, may impose considerable direct and indirect costs including increased health utilization, productivity losses, and greater demands on social insurance systems. In many settings, PCC is not formally recognized as a work-limiting condition, partly because the non-specific and fluctuating nature of its symptoms and lack of biomarkers make it difficult to assess within existing disability frameworks.^30^ This, together with inconsistent use of diagnostic codes,^30^^,^^31^ leaves many affected individuals without adequate support, formally recognized sick leave, or access to workplace accommodations, and in some cases leads to job loss. Greater recognition that PCC, especially its more severe forms, can lead to chronic functional limitations is essential for aligning clinical and disability benefit systems to better support affected individuals. In Switzerland, around 2% of disability applications were reported to be due to PCC.^32^ This aligns well with our estimates that only a small proportion are severely impaired and have significant occupational consequences. However, it may also reflect low recognition of the severe and long-lasting impact of PCC within the current disability assessment framework, which can hinder people from accessing disability benefits despite genuine need and reinforces the importance of establishing clear diagnostic criteria and guidelines.

Strengths of our study include the long follow-up timeframe, population-based approach, large sample size, and good retention rate over three years limiting the extent of bias arising from losses to follow-up. Nevertheless, the following limitations need to be considered. First, people more concerned with their health may have been more likely to participate, and those experiencing PCC may have been more likely to remain in the study. However, while participants were slightly younger on average and less likely to be hospitalised than non-participants,^13^ we found no differences between participants who were retained at the 36-month follow-up and the initial sample. Additionally, we applied inverse probability of censoring weighting and multiple imputation (including with delta adjustment) to at least partially account for differential loss to follow-up, with comparable results across analyses and scenarios. Hence, the direction and magnitude of any potential selection bias is uncertain, and we consider the impact on the association between PCC and work ability to be limited. Second, the participant sample may affect the generalisability of our findings. As this was a population-based study, we included few hospitalised participants. As a result, the applicability of our study to those who experienced very severe COVID-19 may be limited. Similarly, all participants were infected with the ancestral SARS-CoV-2 variant and were not vaccinated at the time of infection. It is unclear how applicable our findings are to later pandemic phases. While newer variants and vaccination have been associated with reduced acute severity and lower risk of PCC, it remains uncertain if the course of PCC and its occupational impact differ across variants. However, milder infections accounted for the majority of SARS-CoV-2 cases, and people infected from the initial wave of the pandemic represent a key group experiencing the highest burden of PCC.^8^ Third, we relied on self-reported measures to assess the presence of PCC and no clinical validation was performed. Given that many PCC symptoms are non-specific and may overlap with comorbid conditions, we cannot exclude some misclassification. Future studies should consider incorporating complementary clinical assessments and other validated screening tools to improve case ascertainment. Along the same lines, work ability was assessed using a subjective instrument and it is unclear if objective measurements would result in the same findings. Nevertheless, self-reporting remains widely used in population-based research and offers key insights to the experiences of affected people. Fourth, we cannot exclude that the lack of improvement in work ability may have been related to other underlying or emerging health conditions or repeated infections that may have worsened their pre-existing PCC or introduced new impairments. Fifth, we did not include a non-infected control group, and we did not have pre-infection baseline work ability. This limits our ability to attribute the observed outcomes directly to SARS-CoV-2 and PCC. Last, we asked participants to report on sick leave and their work performance in the past three years, which could introduce potential recall bias.

In conclusion, in this population-based study with three years of follow-up, we found that individuals affected by PCC reported consistently lower work ability than those without PCC, with little change over time. While some improvements were observed among those with moderate health impairment, persistently low work ability among those with severe health impairment point to a subgroup of people who are at a risk of experiencing longer-term occupational limitations. Recognizing the long-term impact of PCC on individuals’ work capacity and ensuring early access to appropriate rehabilitation services, workplace support measures, as well as adequate financial benefits when necessary, will be essential to mitigate long-term socioeconomic consequences and promote the full recovery and well-being of affected individuals.

## Contributors

TB, DM, JSF, and MAP conceived and planned the Zurich SARS-CoV-2 Cohort study. TB, DM, and MAP coordinated the Zurich SARS-CoV-2 Cohort study. TB, PK, and DM conceived and planned this analysis. TB and DM contributed to participant recruitment and data collection. MAP supervised the project. JSF and MAP obtained funding. TB and DM accessed and verified the data and prepared the analytic datasets. All authors had full access to the data. TB performed the statistical analysis and SH and DM contributed to the statistical analysis. All authors contributed to the interpretation of the findings. TB wrote the draft manuscript. All authors critically revised and provided feedback on the draft manuscript. All authors accept full responsibility for the content of the paper and have seen and approved the final manuscript.

## Data sharing statement

Deidentified individual participant data underlying the findings of this study will be available for researchers submitting a methodologically sound proposal to achieve the aims of the proposal. Proposals should be directed at the corresponding author (Prof. Dr. Milo A. Puhan, miloalan.puhan@uzh.ch).

## Declaration of interests

TB received salary funding by a Moderna Global Fellowship award via institution for work related to the Zurich SARS-CoV-2 Cohort and the Zurich SARS-CoV-2 Vaccine Cohort. PK received salary and project funding via institution by the Swiss National Science Foundation (Grant no 100019M_201,113), the SVA Zurich (Social Insurance Institution of the Canton of Zurich), the Biäsch Foundation, the Foundation Supported Employment CH, and the University of Zurich Foundation. JSF received grants from Gilead Sciences Switzerland, ViiV healthcare, and Merck, as well as an allowance fee from the Swiss Federal Commission for Vaccination Recommendations (EKIF), all unrelated to this work. DM received salary funding by the University of Zurich (UZH) Postdoc Grant (grant No. FK-22-053) via institution for work related to the Zurich SARS-CoV-2 Cohort and the Zurich SARS-CoV-2 Vaccine Cohort. The other authors declare no conflicts of interest.
