# Supplementary material for: Work ability trajectories and sick leave in individuals with post COVID-19 condition: 3-year follow-up of a population-based cohort
Source: Lancet Reg Health Eur. 2025 Nov 20;61:101536. doi: 10.1016/j.lanepe.2025.101536 (PMC12681684; doi:10.1016/j.lanepe.2025.101536)
Supplement: Supplementary Figures and Tables [file mmc1.pdf]

Supplementary Material

Longitudinal work ability trajectories and sick leave in individuals with post COVID-19 condition: 3-year follow-up of a population-based cohort

Tala Ballouz, PhD[1]; Philipp Kerksieck, PhD[1]; Sarah R. Haile, PhD[1]; Holger Dressel, MD[1]; Oliver Hämmig, PhD[1]; Georg F. Bauer, MD[1]; Jan S. Fehr, MD[1]; Milo A. Puhan, PhD[1#]; Dominik Menges, PhD[1]

[1] Epidemiology, Biostatistics and Prevention Institute (EBPI), University of Zurich (UZH), Hirschengraben 84, 8001 Zurich, Switzerland

[#] **Corresponding author**:
Prof. Dr. Milo A. Puhan
Epidemiology, Biostatistics and Prevention Institute (EBPI)
University of Zurich (UZH)
Hirschengraben 84, CH-8001 Zurich, Switzerland
Phone: +41 44 634 46 10, Email: miloalan.puhan@uzh.ch

Supplementary Methods

Details on outcome measurement and definitions.

Outcome	Instrument & question wording	Definition/Categories
**Work ability and sick leave outcomes**		
Current work ability	Work ability index[1–3]<br>• *"Assume that your work ability at its best has a value of 10 points. How many points would you give your current work ability? (0 means that you currently cannot work at all) – (10 work ability at its best)"*	Self-perceived current work ability compared to highest work ability ever (score from 0–10).<br><br>Categorical classification based on von Bonsdorff et al.:[4]<br>• Poor (score ≤6)<br>• Moderate (score 7-8)<br>• Excellent (score ≥9)<br><br>Minimal important difference (MID) proposed to be 2 points based on Stienstra et al. (determined in workers with chronic musculoskeletal pain).[5]
Work ability related to physical demands	Work ability index[1–3]<br>• *"How do you rate your current work ability with respect to the physical demands of your work? (Likert scale from very good to very poor)"*	Self-perceived current work ability in relation to physical demands of work.
Work ability related to mental demands	Work ability index[1–3]<br>• *"How do you rate your current work ability with respect to the mental demands of your work? (Likert scale from very good to very poor)"*	Self-perceived current work ability in relation to mental demands of work.
Estimated future work ability in 2 years	Work ability index[1–3]<br>• *"Do you believe, according to your present state of health, that you will be able to do your current job two years from now? (Unlikely, not certain, relatively certain)"*	Self-perceived estimated work ability in 2 years based on current health status.
Physical performance at work compared to pre-infection levels	• *"How has your physical work ability changed compared to the time before your first coronavirus infection? (Likert scale from greatly worsened to greatly improved)"*<br>• *"Do you attribute the change in your physical work ability to the coronavirus infection? (No, Yes, I don't know)"*	Questions regarding changes in physical performance at work were asked at 36 months after infection.
Physical or mental performance at work compared to pre-infection levels	• *"How has your mental work ability changed compared to the time before your first coronavirus infection? (Likert scale from greatly worsened to greatly improved)"*<br>• *"Do you attribute the change in your mental work ability to the coronavirus infection? (No, Yes, I don't know)"*	Questions regarding changes in mental performance at work were asked at 36 months after infection.
Sick leave related to COVID-19	• *"In the past three years, how long were you on sick leave due to an acute COVID-19 illness or related secondary conditions (e.g., Long COVID, medical complications)? (Never (0 days), 1 day to 1 week, 1 to 2 weeks, 3 to 4 weeks, 1 to 3 months, 4 to 6 months, 6 months to 1 year, more than 1 year ")*	Question regarding sick leave related to COVID-19 was asked at 36 months after infection.

Outcome	Instrument & question wording	Definition/Categories
PCC-related health services	• *Since the last survey (i.e., in the past 6 months), have you used any additional health services due to long COVID, apart from the doctor and hospital visits previously reported?*<br>• *Which additional health services have you used since then? (psychological support and counseling, physiotherapy, occupational therapy, pulmonary/respiratory rehabilitation, general physical rehabilitation, other)*	Question regarding use of PCC-related health services
**Post COVID-19 condition (PCC)**		
Self-reported COVID-19 related symptoms	• *"In the past 7 days, have you had one or more of the following symptoms that are unrelated to a chronic illness or allergy? (Fatigue, Post-exertional malaise, Fever, Cough, Dyspnoea or shortness of breath, Chest pain, Heart palpitations, Altered taste and/or smell, Headache, Concentration difficulties, Memory problems, Vertigo or dizziness, Tremors, Tingling sensation of extremities, Sleep disturbances, Myalgia Arthralgia, Gastrointestinal disturbances, Swallowing difficulties, Hearing problems, Visual disturbances, Skin rash, Hair loss)"*<br>• *"Do you think this symptom is related to or a complication of coronavirus disease "COVID-19"? (No, Yes, I don't know)"*	Presence of at least one symptom in a list of 23 common PCC-related symptoms that were reported by participants to be related to COVID-19.
(Non-)recovery and health impairment	• Self-reported recovery status: *"How do you feel now compared to when you were infected? (fully recovered and symptom free, better but not fully recovered, neither better nor worse, worse)"*<br>• EQ-VAS	1. **Recovered**: participants reporting to fully recovered to their normal health status and symptom-free, or unchanged if asymptomatic at infection.<br>2. **Non-recovered with mild health impairment**: participants reporting not to be back to their normal health status and reporting EQ-VAS scores >70.<br>3. **Non-recovered with moderate health impairment**: participants reporting not to be back to their normal health status and reporting EQ-VAS scores between 51–70.<br>4. **Non-recovered with severe health impairment**: participants reporting not to be back to their normal health status and reporting EQ-VAS scores ≤50.<br><br>EQ-VAS cut-offs were determined based on population-normative values from prior research.[6–9]
Symptom clusters	• Fatigue/physical exertion (defined as presence of fatigue or physical exertion)<br>• Cardiorespiratory (defined as presence of dyspnoea, palpitation, or chest pain)<br>• Neurocognitive (defined as concentration, memory, or sleeping problems)	Presence of self-reported COVID-19 related symptoms belonging to each symptom cluster.

Legend: EQ-VAS, EuroQol visual analogue scale; PCC, post COVID-19 condition.

Multiple imputation

In addition to the primary analysis of IPCW, we used multiple imputation under a missing at random (MAR) assumption to account for missing outcome data. We conducted multivariate imputation by chained equations [1] using the mice R package[10]. The predictor matrix for the incomplete variables included age, sex, body mass index, smoking status, education level, monthly income, comorbidity count, presence of COVID-19 symptoms and (non-)recovery and health impairment (i.e., the same variables as for IPCW). We applied predictive mean matching for continuous variables, logistic regression for binary variables, and polynomial regression for ordered categorical variables. Rubin's rules were used to pool estimates and their variances derived from the robust linear mixed model across the imputed datasets[11]. We further applied the delta adjustment method to assess plausible missing not at random (MNAR) scenarios. Specifically, we shifted the imputed values of the work ability score by a fixed value to reflect potential departures from the MAR assumption. We applied both positive and negative values (delta=+1,+2,-1,-2), while restricting the scale to maximum scores of 10 and minimum scores of 0. The applied deltas corresponded to scenarios where individuals with missing outcome data had better (positive delta; lost to follow-up because they did no longer see value in the study) or worse work ability (negative delta; lost to follow-up because they deteriorated, impeding them from further participation) compared to those without missing data. Again, model estimates and variances were pooled across imputed datasets using Rubin's rule.

References

1 Ilmarinen J. Work ability—a comprehensive concept for occupational health research and prevention. *Scandinavian Journal of Work, Environment & Health* 2009; 35: 1–5.

2 van den Berg TIJ, Elders L a. M, Zwart BCH de, Burdorf A. The effects of work-related and individual factors on the Work Ability Index: a systematic review. *Occupational and Environmental Medicine* 2009; 66: 211–20.

3 Ahlstrom L, Grimby-Ekman A, Hagberg M, Dellve L. The work ability index and single-item question: associations with sick leave, symptoms, and health – a prospective study of women on long-term sick leave. *Scandinavian Journal of Work, Environment & Health* 2010; 36: 404–12.

4 von Bonsdorff MB, Seitsamo J, Ilmarinen J, Nygård C-H, von Bonsdorff ME, Rantanen T. Work ability in midlife as a predictor of mortality and disability in later life: a 28-year prospective follow-up study. *CMAJ* 2011; 183: E235–42.

5 Stienstra M, Edelaar MJA, Fritz B, Reneman MF. Measurement Properties of the Work Ability Score in Sick-Listed Workers with Chronic Musculoskeletal Pain. *J Occup Rehabil* 2022; 32: 103–13.

6 Perneger TV, Combescure C, Courvoisier DS. General Population Reference Values for the French Version of the EuroQol EQ-5D Health Utility Instrument. *Value in Health* 2010; 13: 631–5.

7 Zanini A, Aiello M, Adamo D, *et al.* Estimation of Minimal Clinically Important Difference in EQ-5D Visual Analog Scale Score After Pulmonary Rehabilitation in Subjects With COPD. *Respiratory Care* 2015; 60: 88–95.

8 Wacker ME, Jörres RA, Karch A, *et al.* Assessing health-related quality of life in COPD: comparing generic and disease-specific instruments with focus on comorbidities. *BMC Pulmonary Medicine* 2016; 16: 70.

9 Ballouz T, Menges D, Anagnostopoulos A, *et al.* Recovery and symptom trajectories up to two years after SARS-CoV-2 infection: population based, longitudinal cohort study. *BMJ* 2023; 381: e074425.

10 White IR, Royston P, Wood AM. Multiple imputation using chained equations: Issues and guidance for practice. Statistics in Medicine. 2011;30(4):377–99.

11 Buuren S van, Groothuis-Oudshoorn K. mice: Multivariate Imputation by Chained Equations in R. Journal of Statistical Software. 2011 Dec 12;45:1–67.

Supplementary Results

Supplementary Table 1. Response rates at each of the follow-up timepoints (participants were included based on available data at month 12).

Timepoint	Number (%)
Month 18	552/667 (82.8%)
Month 24	505/667 (75.7%)
Month 30	432/667 (64.8%)
Month 36	416/667 (62.4%)

Supplementary Figure 1. Flow chart of participant enrolment and inclusion in this study.

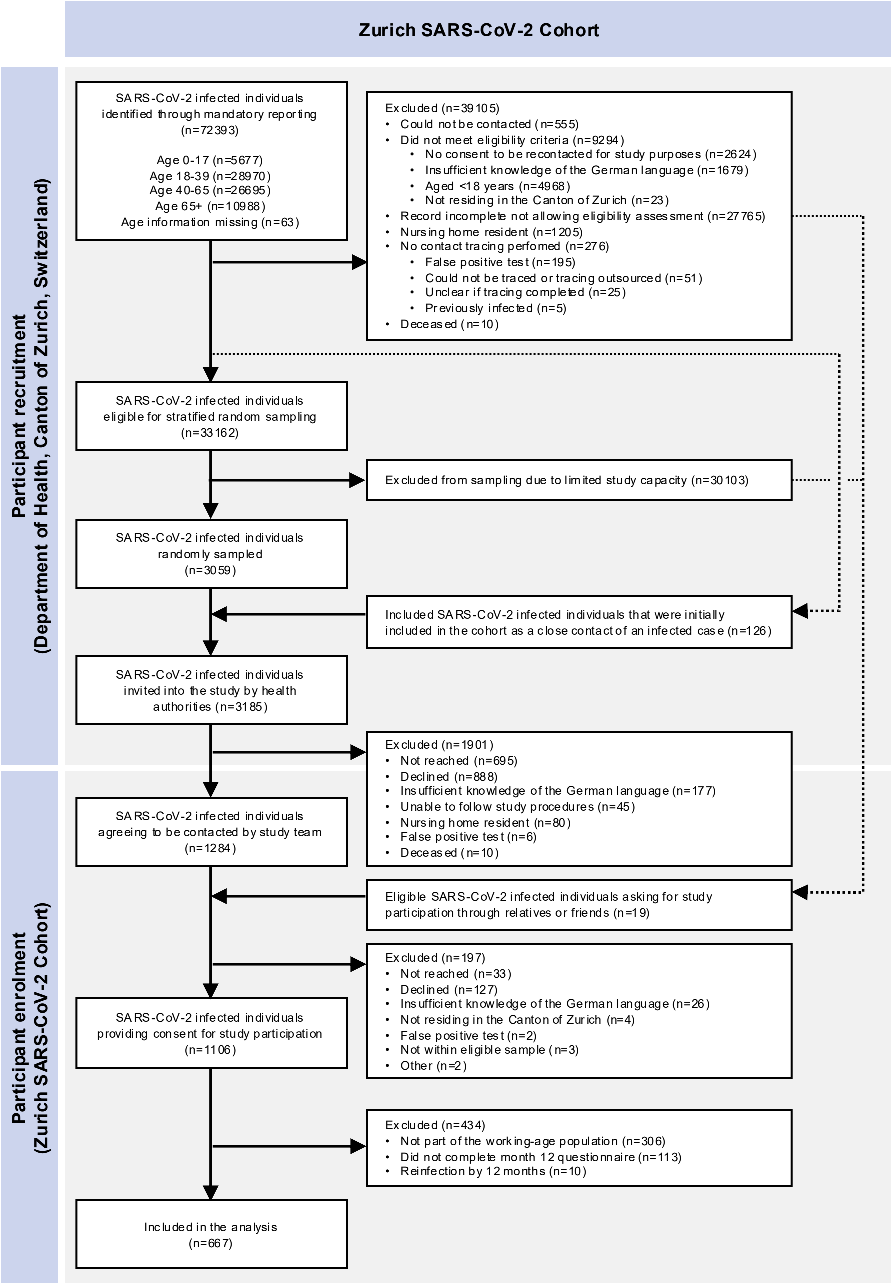

Supplementary Table 2. Detailed study population characteristics, stratified by the presence of self-reported COVID-19 related symptoms and (non-)recovery and health impairment at 12 months after diagnosis of primary infection.

	Self-reported COVID-19 related symptoms		(Non-) recovery and health impairment			
	**No symptoms (N=547)**	**Symptoms (N=120)**	**Recovered (N=562)**	**Mild (N=72)**	**Moderate (N=13)**	**Severe (N=8)**
**Age**						
Median (IQR)	41.0 (30.0 to 51.0)	50.0 (38.0 to 55.0)	41.5 (30.0 to 51.8)	48.0 (35.8 to 55.2)	51.0 (45.0 to 56.0)	53.0 (45.0 to 58.0)
**Age group**						
18-39 years	248 (45.3%)	33 (27.5%)	250 (44.5%)	22 (30.6%)	3 (23.1%)	2 (25.0%)
40-64 years	299 (54.7%)	87 (72.5%)	312 (55.5%)	50 (69.4%)	10 (76.9%)	6 (75.0%)
**Sex**						
Female	283 (51.7%)	79 (65.8%)	292 (52.0%)	47 (65.3%)	11 (84.6%)	6 (75.0%)
Male	264 (48.3%)	41 (34.2%)	270 (48.0%)	25 (34.7%)	2 (15.4%)	2 (25.0%)
**Symptom count at infection**						
Asymptomatic	66 (12.1%)	12 (10.0%)	70 (12.5%)	5 (6.9%)	1 (7.7%)	1 (12.5%)
1-5 symptoms	229 (41.9%)	33 (27.5%)	230 (40.9%)	25 (34.7%)	1 (7.7%)	3 (37.5%)
≥6 symptoms	252 (46.1%)	75 (62.5%)	262 (46.6%)	42 (58.3%)	11 (84.6%)	4 (50.0%)
**Hospitalisation at infection**						
Non-hospitalised	543 (99.3%)	115 (95.8%)	559 (99.5%)	69 (95.8%)	11 (84.6%)	7 (87.5%)
Hospitalised	4 (0.7%)	5 (4.2%)	3 (0.5%)	3 (4.2%)	2 (15.4%)	1 (12.5%)
with ICU stay	0 (0.0%)	1 (0.8%)	0 (0.0%)	0 (0.0%)	1 (7.7%)	0 (0.0%)
**Smoking status**						
Non-smoker	341 (62.6%)	70 (58.3%)	347 (62.0%)	42 (58.3%)	9 (69.2%)	6 (75.0%)
Ex-smoker	121 (22.2%)	33 (27.5%)	127 (22.7%)	20 (27.8%)	2 (15.4%)	1 (12.5%)
Smoker	83 (15.2%)	17 (14.2%)	86 (15.4%)	10 (13.9%)	2 (15.4%)	1 (12.5%)
Missing	*2*	*0*	*2*	*0*	*0*	*0*
**BMI (kg/sqm)**						
Median (IQR)	23.6 (21.4 to 25.9)	24.8 (22.1 to 28.6)	23.6 (21.5 to 26.0)	24.5 (21.7 to 27.0)	30.4 (26.0 to 31.1)	22.3 (20.7 to 23.7)
Missing	*5*	*1*	*6*	*0*	*0*	*0*
**Comorbidity**						
None	449 (82.1%)	79 (65.8%)	460 (81.9%)	49 (68.1%)	6 (46.2%)	4 (50.0%)
1 comorbidity	80 (14.6%)	33 (27.5%)	85 (15.1%)	18 (25.0%)	5 (38.5%)	3 (37.5%)
2+ comorbidities	18 (3.3%)	8 (6.7%)	17 (3.0%)	5 (6.9%)	2 (15.4%)	1 (12.5%)
**History of psychiatric diagnosis**						
No	472 (88.6%)	93 (78.8%)	486 (87.7%)	63 (87.5%)	6 (50.0%)	5 (62.5%)
Yes	61 (11.4%)	25 (21.2%)	68 (12.3%)	9 (12.5%)	6 (50.0%)	3 (37.5%)
Missing	*14*	*2*	*8*	*0*	*1*	*0*
**Education level**						
None or mandatory school	17 (3.1%)	5 (4.2%)	16 (2.9%)	4 (5.6%)	0 (0.0%)	0 (0.0%)
Vocational training or specialized baccalaureate	192 (35.1%)	55 (46.6%)	199 (35.5%)	33 (46.5%)	7 (53.8%)	3 (37.5%)
Higher technical school or college	164 (30.0%)	29 (24.6%)	173 (30.8%)	16 (22.5%)	2 (15.4%)	2 (25.0%)
University	174 (31.8%)	29 (24.6%)	173 (30.8%)	18 (25.4%)	4 (30.8%)	3 (37.5%)
Missing	*0*	*2*	*1*	*1*	*0*	*0*
**Employment at infection**						
Employed or self-employed	483 (88.3%)	99 (82.5%)	494 (87.9%)	61 (84.7%)	11 (84.6%)	4 (50.0%)

	Self-reported COVID-19 related symptoms		(Non-) recovery and health impairment			
	**No symptoms (N=547)**	**Symptoms (N=120)**	**Recovered (N=562)**	**Mild (N=72)**	**Moderate (N=13)**	**Severe (N=8)**
Student	42 (7.7%)	4 (3.3%)	45 (8.0%)	1 (1.4%)	0 (0.0%)	0 (0.0%)
Housewife/family manager	9 (1.6%)	1 (0.8%)	9 (1.6%)	1 (1.4%)	0 (0.0%)	0 (0.0%)
Unemployed	9 (1.6%)	10 (8.3%)	10 (1.8%)	7 (9.7%)	1 (7.7%)	1 (12.5%)
Disability insurance benefits	0 (0.0%)	4 (3.3%)	0 (0.0%)	0 (0.0%)	1 (7.7%)	3 (37.5%)
Other	4 (0.7%)	2 (1.7%)	4 (0.7%)	2 (2.8%)	0 (0.0%)	0 (0.0%)
**Monthly household income**						
<6'000 CHF	148 (28.0%)	40 (34.8%)	156 (28.7%)	23 (32.9%)	3 (25.0%)	4 (50.0%)
6'000 - 12'000 CHF	229 (43.3%)	52 (45.2%)	234 (43.1%)	33 (47.1%)	7 (58.3%)	1 (12.5%)
>12'000 CHF	152 (28.7%)	23 (20.0%)	153 (28.2%)	14 (20.0%)	2 (16.7%)	3 (37.5%)
Missing	*18*	*5*	*19*	*2*	*1*	*0*
**Nationality**						
Swiss	462 (84.5%)	97 (80.8%)	479 (85.2%)	56 (77.8%)	9 (69.2%)	7 (87.5%)
Non-Swiss	85 (15.5%)	23 (19.2%)	83 (14.8%)	16 (22.2%)	4 (30.8%)	1 (12.5%)

Legend: IQR, interquartile range; SD, standard deviation; CHF, Swiss Francs

Supplementary Table 3. Work ability scores over time, stratified by presence of self-reported COVID-19 related symptoms and (non-)recovery and health impairment at 12 months after diagnosis of primary infection, in the main and sensitivity analyses.

		IPCW			Available case (unweighted)			Complete case (unweighted)		
**Outcome**	**Timepoint**	**N**	**Mean (SD)**	**Median (IQR)**	**N**	**Mean (SD)**	**Median (IQR)**	**N**	**Mean (SD)**	**Median (IQR)**
**Symptoms**										
No Symptoms	M12	547	8.86 (1.54)	9 (8–10)	537	8.86 (1.54)	9 (8–10)	299	8.95 (1.42)	9 (8–10)
	M18	547	8.97 (1.43)	9 (8–10)	442	8.96 (1.43)	9 (8–10)	299	8.98 (1.32)	9 (8–10)
	M24	547	8.99 (1.29)	9 (8–10)	397	8.98 (1.32)	9 (8–10)	299	8.99 (1.30)	9 (9–10)
	M30	547	8.96 (1.40)	9 (9–10)	342	8.95 (1.41)	9 (9–10)	299	8.97 (1.35)	9 (9–10)
	M36	547	8.78 (1.46)	9 (8–10)	315	8.78 (1.47)	9 (8–10)	299	8.80 (1.40)	9 (8–10)
Symptoms	M12	120	7.88 (2.21)	8 (7–9)	119	7.88 (2.21)	8 (7–9)	70	7.94 (2.33)	9 (8–9)
	M18	120	7.57 (2.38)	8 (7–9)	105	7.62 (2.34)	8 (7–9)	70	7.74 (2.34)	8 (7–9)
	M24	120	7.50 (2.59)	8 (7–9)	98	7.62 (2.43)	8 (7–9)	70	7.66 (2.43)	8 (7–9)
	M30	120	7.25 (2.94)	9 (6–9)	85	7.41 (2.81)	9 (6–9)	70	7.43 (2.84)	8.5 (6–9.75)
	M36	120	7.55 (2.32)	8 (7–9)	76	7.58 (2.26)	8 (7–9)	70	7.53 (2.33)	8 (7–9)
**(Non-) recovery and health impairment**										
Recovered	M12	562	8.93 (1.43)	9 (8–10)	555	8.93 (1.43)	9 (8–10)	310	9.04 (1.28)	9 (9–10)
	M18	562	8.98 (1.37)	9 (8–10)	456	8.97 (1.38)	9 (8–10)	310	9.02 (1.23)	9 (8–10)
	M24	562	8.98 (1.30)	9 (8–10)	410	8.99 (1.31)	9 (8–10)	310	9.01 (1.26)	9 (9–10)
	M30	562	8.95 (1.46)	9 (9–10)	354	8.95 (1.47)	9 (9–10)	310	8.97 (1.41)	9 (9–10)
	M36	562	8.80 (1.42)	9 (8–10)	330	8.79 (1.42)	9 (8–10)	310	8.82 (1.35)	9 (8–10)
Mild	M12	72	8.36 (1.04)	8 (8–9)	72	8.36 (1.04)	8 (8–9)	42	8.4 (0.96)	8 (8–9)
	M18	72	8.24 (1.61)	8 (8–9)	65	8.25 (1.57)	8 (8–9)	42	8.24 (1.41)	8 (8–9)
	M24	72	8.47 (1.14)	9 (8–9)	60	8.50 (1.13)	9 (8–9)	42	8.36 (1.16)	9 (8–9)
	M30	72	8.04 (1.95)	8 (7–10)	49	8.10 (1.97)	9 (7–10)	42	7.95 (2.01)	8 (7–9)
	M36	72	7.86 (1.86)	8 (7–9)	43	7.93 (1.86)	8 (7–9)	42	7.95 (1.87)	8 (7–9)
Moderate	M12	13	4.92 (2.10)	5 (4–6)	13	4.92 (2.10)	5 (4–6)	10	4.60 (1.96)	4.5 (3.25–6)
	M18	13	5.11 (2.13)	6 (3–6)	12	5.25 (2.22)	6 (3.75–6)	10	5.60 (2.27)	6 (5.25–6)
	M24	13	4.21 (2.20)	4 (2–6)	11	4.45 (2.11)	4 (3–6)	10	4.50 (2.22)	5 (3–6)
	M30	13	5.53 (2.79)	5 (4–8)	12	5.58 (2.57)	5 (4–7.25)	10	5.50 (2.68)	5 (4.25–7)
	M36	13	6.23 (2.26)	7 (6–8)	11	6.45 (1.97)	7 (6–7.5)	10	6.40 (2.07)	7 (6–7.75)
Severe	M12	8	3.38 (2.93)	3 (0–5)	8	3.38 (2.92)	3.5 (0.75–5.25)	4	3.25 (3.59)	2.5 (0.75–5)
	M18	8	2.87 (3.56)	2 (0–6)	7	3.00 (3.51)	2 (0–5)	4	2.75 (4.27)	1 (0–3.75)
	M24	8	2.60 (3.70)	2 (0–6)	7	3.00 (3.70)	2 (0.5–4)	4	3.25 (4.57)	1.5 (0.75–4)
	M30	8	2.74 (3.52)	1 (1–3)	7	3.29 (3.77)	1 (1–5)	4	3.00 (4.69)	1 (0.75–3.25)
	M36	8	2.49 (3.22)	1 (0–7)	4	2.50 (3.11)	1.5 (0.75–3.25)	4	2.5 (3.11)	1.5 (0.75–3.25)

Legend: IPCW, inverse probability of censoring weighting; N, number; IQR, interquartile range; SD, standard deviation; M, month

Supplementary Table 4. Results from regular and robust linear mixed effects models for the association of current work ability (as continuous variable) and presence of COVID-19 related symptoms at 12 months after infection. Time (in years), COVID-19 related symptoms and their interaction are included as fixed-effect predictors. This can be interpreted as the difference in trends over time relative to the comparison group (i.e., whether there is a worsening or improvement over time beyond what would be expected in a population without post COVID-19 condition). Models were further adjusted for age, sex, education status, baseline EuroQoL visual analogue scale (EQ-VAS), comorbidity count, prior psychiatric diagnosis, and hospitalisation due to COVID-19 with a random intercept to account for within-person correlation. The primary analysis included stabilized inverse probability of censoring weighting (IPCW) to account for potential selection bias introduced by missing data due to losses to follow-up. Multiple imputation-based models without and with delta adjustment were incorporated as sensitivity analyses to test the robustness of findings under missingness at random (MAR) and plausible missingness not at random (MNAR) scenarios. Confidence intervals for IPCW analyses are based on boostrapping (n=1000), while those for multiple imputation analyses were derived by applying Rubin's rule to pool model-derived estimates and variances across imputations for computational reasons. Confidence intervals for multiple imputation analyses are thus less robust, not directly comparable with IPCW, and should be interpreted with caution.

	**Regular linear mixed effects model (IPCW)**	***Robust linear mixed effects model (IPCW)***	**Robust linear mixed effects model (MI, no delta adjustment)**	**Robust linear mixed effects model (MI, delta=+1)**	**Robust linear mixed effects model (MI, delta=+2)**	**Robust linear mixed effects model (MI, delta=-1)**	**Robust linear mixed effects model (MI, delta=-2)**
Term	***Coefficient (95% CI)***	***Coefficient (95% CI)***	***Coefficient (95% CI)***	***Coefficient (95% CI)***	***Coefficient (95% CI)***	***Coefficient (95% CI)***	***Coefficient (95% CI)***
**Timepoint (per year increase)**	-0.06 (-0.13 to 0.02)	-0.04 (-0.13 to 0.02)	-0.03 (-0.10 to 0.03)	0.07 (0.02 to 0.13)	0.12 (0.08 to 0.17)	-0.21 (-0.28 to -0.15)	-0.39 (-0.46 to -0.33)
No symptoms	Ref.	Ref.	Ref.	Ref.	Ref.	Ref.	Ref.
Symptoms	-0.67 (-1.16 to -0.25)	-0.68 (-1.16 to -0.25)	-0.86 (-1.19 to -0.52)	-0.87 (-1.19 to -0.55)	-0.90 (-1.21 to -0.58)	-0.88 (-1.22 to -0.53)	-0.88 (-1.27 to -0.50)
**Age at infection (per year increase)**	-0.00 (-0.01 to 0.01)	-0.00 (-0.01 to 0.01)	0.00 (-0.01 to 0.01)	-0.00 (-0.01 to 0.00)	-0.01 (-0.01 to 0.00)	0.01 (-0.00 to 0.01)	0.01 (0.00 to 0.02)
**Sex**							
Female	Ref.	Ref.	Ref.	Ref.	Ref.	Ref.	Ref.
Male	0.09 (-0.11 to 0.27)	0.06 (-0.11 to 0.27)	0.07 (-0.10 to 0.24)	0.08 (-0.09 to 0.25)	0.08 (-0.09 to 0.25)	0.06 (-0.11 to 0.24)	0.05 (-0.15 to 0.24)
**Comorbidity count**							
0-1 comorbidity	Ref.	Ref.	Ref.	Ref.	Ref.	Ref.	Ref.
2+ comorbidities	-0.49 (-1.37 to 0.32)	-0.42 (-1.37 to 0.32)	-0.25 (-0.71 to 0.20)	-0.16 (-0.61 to 0.28)	-0.12 (-0.56 to 0.32)	-0.39 (-0.86 to 0.08)	-0.53 (-1.04 to -0.01)
**Hospitalisation at infection**							
Hospitalized	Ref.	Ref.	Ref.	Ref.	Ref.	Ref.	Ref.
Non-hospitalized	1.06 (-0.08 to 2.59)	1.12 (-0.08 to 2.59)	1.01 (0.28 to 1.73)	1.01 (0.29 to 1.73)	1.02 (0.30 to 1.74)	1.01 (0.27 to 1.76)	1.01 (0.18 to 1.84)
**Baseline EuroQoL visual analogue scale (per 1 point increase)**	0.04 (0.02 to 0.05)	0.04 (0.02 to 0.05)	0.02 (0.02 to 0.03)	0.02 (0.01 to 0.03)	0.02 (0.01 to 0.03)	0.03 (0.02 to 0.03)	0.03 (0.02 to 0.04)
**Education level**							
None or mandatory school	Ref.	Ref.	Ref.	Ref.	Ref.	Ref.	Ref.
Vocational training or specialized baccalaureate	-0.06 (-0.64 to 0.55)	-0.04 (-0.64 to 0.55)	0.22 (-0.34 to 0.78)	0.10 (-0.44 to 0.64)	0.03 (-0.49 to 0.55)	0.35 (-0.22 to 0.92)	0.49 (-0.13 to 1.10)
Higher technical school or college	-0.42 (-0.99 to 0.15)	-0.33 (-0.99 to 0.15)	0.01 (-0.56 to 0.59)	-0.09 (-0.64 to 0.46)	-0.15 (-0.68 to 0.38)	0.15 (-0.44 to 0.73)	0.27 (-0.36 to 0.91)
University	-0.19 (-0.76 to 0.39)	-0.18 (-0.76 to 0.39)	0.03 (-0.53 to 0.58)	-0.11 (-0.65 to 0.42)	-0.19 (-0.71 to 0.33)	0.21 (-0.35 to 0.78)	0.41 (-0.21 to 1.03)

**History of psychiatric diagnosis**							
No	Ref.	Ref.	Ref.	Ref.	Ref.	Ref.	Ref.
Yes	-0.89 (-1.33 to -0.48)	-0.79 (-1.33 to -0.48)	-0.55 (-0.81 to -0.29)	-0.56 (-0.82 to -0.30)	-0.56 (-0.82 to -0.30)	-0.51 (-0.78 to -0.25)	-0.49 (-0.78 to -0.19)
**Timepoint * Self-reported COVID-19 related symptoms (per year increase)**							
Timepoint * No symptoms	Ref.	Ref.	Ref.	Ref.	Ref.	Ref.	Ref.
Timepoint * Symptoms	-0.12 (-0.29 to 0.07)	-0.12 (-0.29 to 0.07)	-0.06 (-0.20 to 0.07)	-0.05 (-0.17 to 0.08)	-0.01 (-0.13 to 0.10)	-0.03 (-0.17 to 0.11)	-0.00 (-0.15 to 0.15)

Legend: CI, confidence interval; Ref, reference level; IPCW: inverse probability of censoring weighting; MI: multiple imputation

Supplementary Table 5. Results from regular and robust linear mixed effects models for the association of current work ability (as continuous variable) and (non-)recovery and health impairment at 12 months after infection. Time (in years), health impairment status and their interaction are included as fixed-effect predictors. This can be interpreted as the difference in trends over time relative to the comparison group (i.e., whether there is a worsening or improvement over time beyond what would be expected in a population without post COVID-19 condition). Models were further adjusted for age, sex, education status, baseline EuroQoL visual analogue scale (EQ-VAS), comorbidity count, prior psychiatric diagnosis, and hospitalisation due to COVID-19 with a random intercept to account for within-person correlation. The primary analysis included stabilized inverse probability of censoring weighting (IPCW) to account for potential selection bias introduced by missing data due to losses to follow-up. Multiple imputation-based models without and with delta adjustment were incorporated as sensitivity analyses to test the robustness of findings under missingness at random (MAR) and plausible missingness not at random (MNAR) scenarios. Confidence intervals for IPCW analyses are based on boostrapping (n=1000), while those for multiple imputation analyses were derived by applying Rubin's rule to pool model-derived estimates and variances across imputations for computational reasons. Confidence intervals for multiple imputation analyses are thus less robust, not directly comparable with IPCW, and should be interpreted with caution.

	**Regular linear mixed effects model (IPCW)**	***Robust linear mixed effects model (IPCW)***	**Robust linear mixed effects model (MI, no delta adjustment)**	**Robust linear mixed effects model (MI, delta=+1)**	**Robust linear mixed effects model (MI, delta=+2)**	**Robust linear mixed effects model (MI, delta=-1)**	**Robust linear mixed effects model (MI, delta=-2)**
Term	***Coefficient (95% CI)***	***Coefficient (95% CI)***	***Coefficient (95% CI)***	***Coefficient (95% CI)***	***Coefficient (95% CI)***	***Coefficient (95% CI)***	***Coefficient (95% CI)***
**Timepoint (per year increase)**	-0.10 (-0.17 to -0.02)	-0.07 (-0.17 to -0.02)	-0.05 (-0.11 to 0.01)	0.06 (0.00 to 0.11)	0.11 (0.06 to 0.15)	-0.23 (-0.29 to -0.17)	-0.41 (-0.47 to -0.34)
**(Non-)recovery**							
Recovered	Ref.	Ref.	Ref.	Ref.	Ref.	Ref.	Ref.
Mild health impairment	-0.50 (-0.85 to -0.17)	-0.55 (-0.85 to -0.17)	-0.58 (-0.97 to -0.18)	-0.66 (-1.03 to -0.28)	-0.70 (-1.06 to -0.34)	-0.50 (-0.91 to -0.09)	-0.41 (-0.86 to 0.05)
Moderate health impairment	-4.40 (-5.95 to -2.86)	-4.45 (-5.95 to -2.86)	-4.43 (-5.36 to -3.50)	-4.39 (-5.29 to -3.48)	-4.36 (-5.28 to -3.45)	-4.48 (-5.43 to -3.52)	-4.48 (-5.53 to -3.42)
Severe health impairment	-5.23 (-6.88 to -3.51)	-5.44 (-6.88 to -3.51)	-5.18 (-6.40 to -3.97)	-5.27 (-6.47 to -4.08)	-5.32 (-6.49 to -4.15)	-5.18 (-6.41 to -3.95)	-5.18 (-6.54 to -3.83)
**Age at infection (per year increase)**	0.00 (-0.01 to 0.01)	0.00 (-0.01 to 0.01)	0.00 (-0.00 to 0.01)	-0.00 (-0.01 to 0.00)	-0.00 (-0.01 to 0.00)	0.01 (0.00 to 0.01)	0.01 (0.01 to 0.02)
**Sex**							
Female	Ref.	Ref.	Ref.	Ref.	Ref.	Ref.	Ref.
Male	0.01 (-0.16 to 0.19)	-0.01 (-0.16 to 0.19)	0.01 (-0.14 to 0.16)	0.02 (-0.12 to 0.16)	0.02 (-0.12 to 0.17)	0.00 (-0.15 to 0.15)	-0.01 (-0.19 to 0.16)
**Comorbidity count**							
0-1 comorbidity	Ref.	Ref.	Ref.	Ref.	Ref.	Ref.	Ref.
2+ comorbidities	-0.35 (-1.16 to 0.29)	-0.22 (-1.16 to 0.29)	-0.12 (-0.51 to 0.27)	-0.03 (-0.41 to 0.35)	0.01 (-0.37 to 0.39)	-0.25 (-0.65 to 0.16)	-0.38 (-0.84 to 0.08)
**Hospitalisation at infection**							
Hospitalized	Ref.	Ref.	Ref.	Ref.	Ref.	Ref.	Ref.
Non-hospitalized	0.27 (-1.3 to 1.68)	0.31 (-1.3 to 1.68)	0.10 (-0.53 to 0.73)	0.07 (-0.55 to 0.70)	0.08 (-0.55 to 0.70)	0.16 (-0.50 to 0.81)	0.23 (-0.52 to 0.98)
**Baseline EuroQoL visual analogue scale (per 1 point increase)**	0.03 (0.02 to 0.04)	0.03 (0.02 to 0.04)	0.02 (0.01 to 0.03)	0.02 (0.01 to 0.02)	0.02 (0.01 to 0.02)	0.02 (0.02 to 0.03)	0.03 (0.02 to 0.03)
**Education level**							
None or mandatory school	Ref.	Ref.	Ref.	Ref.	Ref.	Ref.	Ref.
Vocational training or specialized baccalaureate	0.13 (-0.42 to 0.77)	0.16 (-0.42 to 0.77)	0.37 (-0.12 to 0.86)	0.25 (-0.20 to 0.71)	0.18 (-0.26 to 0.62)	0.50 (-0.00 to 1.01)	0.64 (0.08 to 1.20)

Higher technical school or college	-0.24 (-0.79 to 0.4)	-0.11 (-0.79 to 0.4)	0.16 (-0.34 to 0.65)	0.05 (-0.41 to 0.52)	-0.01 (-0.46 to 0.43)	0.30 (-0.22 to 0.81)	0.43 (-0.14 to 1.00)
University	0.05 (-0.48 to 0.66)	0.07 (-0.48 to 0.66)	0.25 (-0.24 to 0.73)	0.11 (-0.35 to 0.56)	0.02 (-0.42 to 0.46)	0.44 (-0.07 to 0.94)	0.63 (0.07 to 1.18)
**History of psychiatric diagnosis**							
No	Ref.	Ref.	Ref.	Ref.	Ref.	Ref.	Ref.
Yes	-0.75 (-1.08 to -0.46)	-0.64 (-1.08 to -0.46)	-0.48 (-0.70 to -0.26)	-0.50 (-0.71 to -0.28)	-0.50 (-0.72 to -0.28)	-0.44 (-0.67 to -0.21)	-0.40 (-0.66 to -0.14)
**Timepoint * (non-)recovery and health impairment (per year increase)**							
Timepoint * Recovered	Ref.	Ref.	Ref.	Ref.	Ref.	Ref.	Ref.
Timepoint * Mild health impairment	-0.02 (-0.21 to 0.18)	-0.00 (-0.21 to 0.18)	-0.01 (-0.18 to 0.16)	0.03 (-0.12 to 0.18)	0.07 (-0.07 to 0.21)	-0.02 (-0.19 to 0.15)	-0.04 (-0.23 to 0.14)
Timepoint * Moderate health impairment	0.76 (-0.04 to 1.46)	0.72 (-0.04 to 1.46)	0.69 (0.31 to 1.07)	0.63 (0.27 to 0.99)	0.63 (0.28 to 0.97)	0.80 (0.42 to 1.18)	0.89 (0.48 to 1.30)
Timepoint * Severe health impairment	-0.02 (-0.4 to 0.33)	-0.00 (-0.4 to 0.33)	-0.03 (-0.56 to 0.49)	0.01 (-0.47 to 0.50)	0.08 (-0.39 to 0.54)	0.03 (-0.51 to 0.56)	0.12 (-0.46 to 0.70)

Legend: CI, confidence interval; Ref, reference level; IPCW: inverse probability of censoring weighting; MI: multiple imputation

Supplementary Table 6. Results from regular and robust linear mixed effects models for the association of current work ability (as continuous variable) and presence of COVID-19 related symptoms at 12 months after infection, among participants who reported being employed or self-employed at baseline. Time (in years), COVID-19 related symptoms and their interaction are included as fixed-effect predictors. This can be interpreted as the difference in trends over time relative to the comparison group (i.e., whether there is a worsening or improvement over time beyond what would be expected in a population without post COVID-19 condition). Models were further adjusted for age, sex, education status, baseline EuroQoL visual analogue scale (EQ-VAS), comorbidity count, prior psychiatric diagnosis, and hospitalisation due to COVID-19 with a random intercept to account for within-person correlation. Stabilized inverse probability of censoring weighting (IPCW) was applied to account for potential selection bias introduced by missing data due to losses to follow-up.

	**Regular linear mixed effects model**	**Robust linear mixed effects model**
Term	***Coefficient (95% CI)***	***Coefficient (95% CI)***
**Timepoint (per year increase)**	-0.04 (-0.12 to 0.04)	-0.03 (-0.12 to 0.04)
**Self-reported COVID-19 related symptoms at 12 month**		
No symptoms	Ref.	Ref.
Symptoms	-0.45 (-0.95 to 0.04)	-0.49 (-0.95 to 0.04)
**Age at infection (per year increase)**	0.00 (-0.01 to 0.01)	0.00 (-0.01 to 0.01)
**Sex**		
Female	Ref.	Ref.
Male	0.07 (-0.15 to 0.27)	0.05 (-0.15 to 0.27)
**Comorbidity count**		
0-1 comorbidity	Ref.	Ref.
2+ comorbidities	-0.31 (-1.09 to 0.37)	-0.18 (-1.09 to 0.37)
**Hospitalisation at infection**		
Hospitalized	Ref.	Ref.
Non-hospitalized	1.33 (0.18 to 2.87)	1.40 (0.18 to 2.87)
**Baseline EuroQoL visual analogue scale (per 1 point increase)**	0.04 (0.03 to 0.06)	0.04 (0.03 to 0.06)
**Education level**		
None or mandatory school	Ref.	Ref.
Vocational training or specialized baccalaureate	-0.23 (-0.82 to 0.33)	-0.17 (-0.82 to 0.33)
Higher technical school or college	-0.55 (-1.14 to 0.03)	-0.41 (-1.14 to 0.03)
University	-0.40 (-0.98 to 0.17)	-0.34 (-0.98 to 0.17)
**History of psychiatric diagnosis**		
No	Ref.	Ref.
Yes	-0.69 (-1.09 to -0.37)	-0.54 (-1.09 to -0.37)
**Timepoint * Self-reported COVID-19 related symptoms (per year increase)**		
Timepoint * No symptoms	Ref.	Ref.
Timepoint * Symptoms	-0.13 (-0.34 to 0.08)	-0.12 (-0.34 to 0.08)

Legend: CI, confidence interval; Ref, reference level

Supplementary Table 7. Results from robust linear mixed effects models for the association of current work ability (as continuous variable) and three main symptom clusters (fatigue/physical exertion, cardiorespiratory, or neurocognitive) at 12 months after infection. Separate models were run for each of the clusters. Time (in years), symptom cluster and their interaction are included as fixed-effect predictors. This can be interpreted as the difference in trends over time relative to the comparison group (i.e., whether there is a worsening or improvement over time beyond what would be expected in a population without the symptom cluster). Models were further adjusted for age, sex, education status, baseline EuroQoL visual analogue scale (EQ-VAS), comorbidity count, prior psychiatric diagnosis, and hospitalisation due to COVID-19 with a random intercept to account for within-person correlation. Stabilized inverse probability of censoring weighting (IPCW) was applied to account for potential selection bias introduced by missing data due to losses to follow-up.

	**Fatigue/Physical exterion symptoms**	**Cardiorespiratory symptoms**	**Neurocognitive symptoms**
Term	***Coefficient (95% CI)***	***Coefficient (95% CI)***	***Coefficient (95% CI)***
**Timepoint**	-0.06 (-0.15 to 0.00)	-0.07 (-0.16 to -0.01)	-0.05 (-0.15 to 0.00)
**Presence of symptom cluster at 12 months**			
No symptom cluster	Ref.	Ref.	Ref.
Symptom cluster	-1.65 (-2.55 to -0.90)	-1.99 (-3.12 to -0.82)	-1.14 (-2.21 to -0.24)
**Age at infection (per year increase)**	-0.00 (-0.01 to 0.01)	-0.00 (-0.01 to 0.01)	-0.00 (-0.01 to 0.01)
**Sex**			
Female	Ref.	Ref.	Ref.
Male	0.06 (-0.09 to 0.27)	0.02 (-0.15 to 0.25)	0.07 (-0.10 to 0.3)
**Comorbidity count**			
0-1 comorbidity	Ref.	Ref.	Ref.
2+ comorbidities	-0.33 (-1.49 to 0.47)	-0.41 (-1.38 to 0.33)	-0.55 (-1.66 to 0.24)
**Hospitalisation at infection**			
Hospitalized	Ref.	Ref.	Ref.
Non-hospitalized	0.62 (-0.79 to 1.96)	1.21 (-0.28 to 2.99)	1.16 (-0.13 to 2.36)
**Baseline EuroQoL visual analog scale (per 1 point change)**	0.04 (0.02 to 0.05)	0.04 (0.02 to 0.05)	0.04 (0.03 to 0.05)
**Education level**			
None or mandatory school	Ref.	Ref.	Ref.
Vocational training or specialized baccalaureate	-0.08 (-0.74 to 0.54)	-0.11 (-0.72 to 0.40)	-0.03 (-0.72 to 0.58)
Higher technical school or college	-0.35 (-1.10 to 0.18)	-0.36 (-1.09 to 0.14)	-0.25 (-1.03 to 0.27)
University	-0.20 (-0.82 to 0.42)	-0.21 (-0.84 to 0.33)	-0.10 (-0.80 to 0.49)
**History of psychiatric diagnosis**			
No	Ref.	Ref.	Ref.
Yes	-0.76 (-1.27 to -0.46)	-0.82 (-1.35 to -0.50)	-0.79 (-1.30 to -0.49)
**Timepoint * Presence of symptom cluster (per year change)**			
Timepoint * No symptom cluster	Ref.	Ref.	Ref.
Timepoint * Symptom cluster	-0.06 (-0.40 to 0.30)	0.16 (-0.26 to 0.59)	-0.18 (-0.58 to 0.29)

Legend: CI, confidence interval; Ref, reference level

Supplementary Table 8. Results from robust linear mixed effects models for the association of current work ability and presence of COVID-19 related symptoms at 12 months after infection within subgroups based on age group, sex, comorbidity count, and history of psychiatric diagnosis. Stabilized inverse probability of censoring weighting (IPCW) was applied to account for potential selection bias introduced by missing data due to losses to follow-up.

Interaction	Difference in scores* (95% CI)	Difference in change in scores* (95% CI)
**Male vs female**		
Female	-0.72 (-1.35 to -0.19)	-0.20 (-0.4 to 0.01)
Male	-0.60 (-1.15 to -0.09)	-0.01 (-0.18 to 0.17)
Difference	*0.11 (-0.62 to 0.95)*	*0.19 (-0.07 to 0.47)*
**40-64 years vs 18-39 years**		
18-39 years	-0.30 (-1.11 to 0.47)	-0.05 (-0.37 to 0.23)
40-64 years	-0.86 (-1.38 to -0.38)	-0.13 (-0.29 to 0.03)
Difference	*-0.56 (-1.44 to 0.39)*	*-0.08 (-0.41 to 0.28)*
**≥2 comorbidities vs 0-1 comorbidity**[†]		
0-1 comorbidity	-0.61 (-1.04 to -0.25)	-0.14 (-0.29 to 0.01)
≥2 comorbidities	-1.78 (-5.47 to 1.12)	-0.01 (-0.74 to 0.8)
Difference	*-1.17 (-5.01 to 1.72)*	*0.13 (-0.64 to 0.93)*
**History of psychiatric diagnosis vs no history of psychiatric diagnosis**		
No history of psychiatric diagnosis	-0.38 (-0.8 to -0.01)	-0.18 (-0.32 to -0.02)
History of psychiatric diagnosis	-2.10 (-3.58 to -0.77)	0.11 (-0.32 to 0.53)
Difference	*-1.72 (-3.26 to -0.32)*	*0.28 (-0.17 to 0.75)*

Legend: CI, confidence interval. * Subgroup estimates are interpreted as the difference in mean scores or the difference in the change in scores over time for the comparison between individuals with self-reported COVID-19 related symptoms compared to those without symptoms within the respective subgroups, while difference in difference estimates quantify the extent of effect modification for the respective stratification variable. We used robust linear mixed effect including an interaction term for the respective stratification variable and adjusted for age, sex, education status, baseline EuroQol visual analogue scale (EQ-VAS), comorbidity count, history of psychiatric diagnosis, and hospitalisation due to COVID-19. [†] Comorbidities were assessed as any of the following: hypertension, diabetes, cardiovascular disease, chronic respiratory disease, chronic kidney disease, past or present malignancy, or immune suppression

Supplementary Figure 2. Average work ability scores over time by presence of COVID-19 related symptoms at 12 months within subgroups based on age (panel a), sex (panel b), comorbidity count (panel c), and history of psychiatric diagnosis (panel d). Average scores were estimated using robust linear mixed effects models analogous to the primary analysis while including an interaction term for the respective stratification variable and adjusting for age, sex, education level, comorbidity count, history of psychiatric diagnosis, hospitalisation status during acute infection, and baseline health status (EQ-VAS). Shaded areas represent 95% confidence intervals derived using bootstrapping.

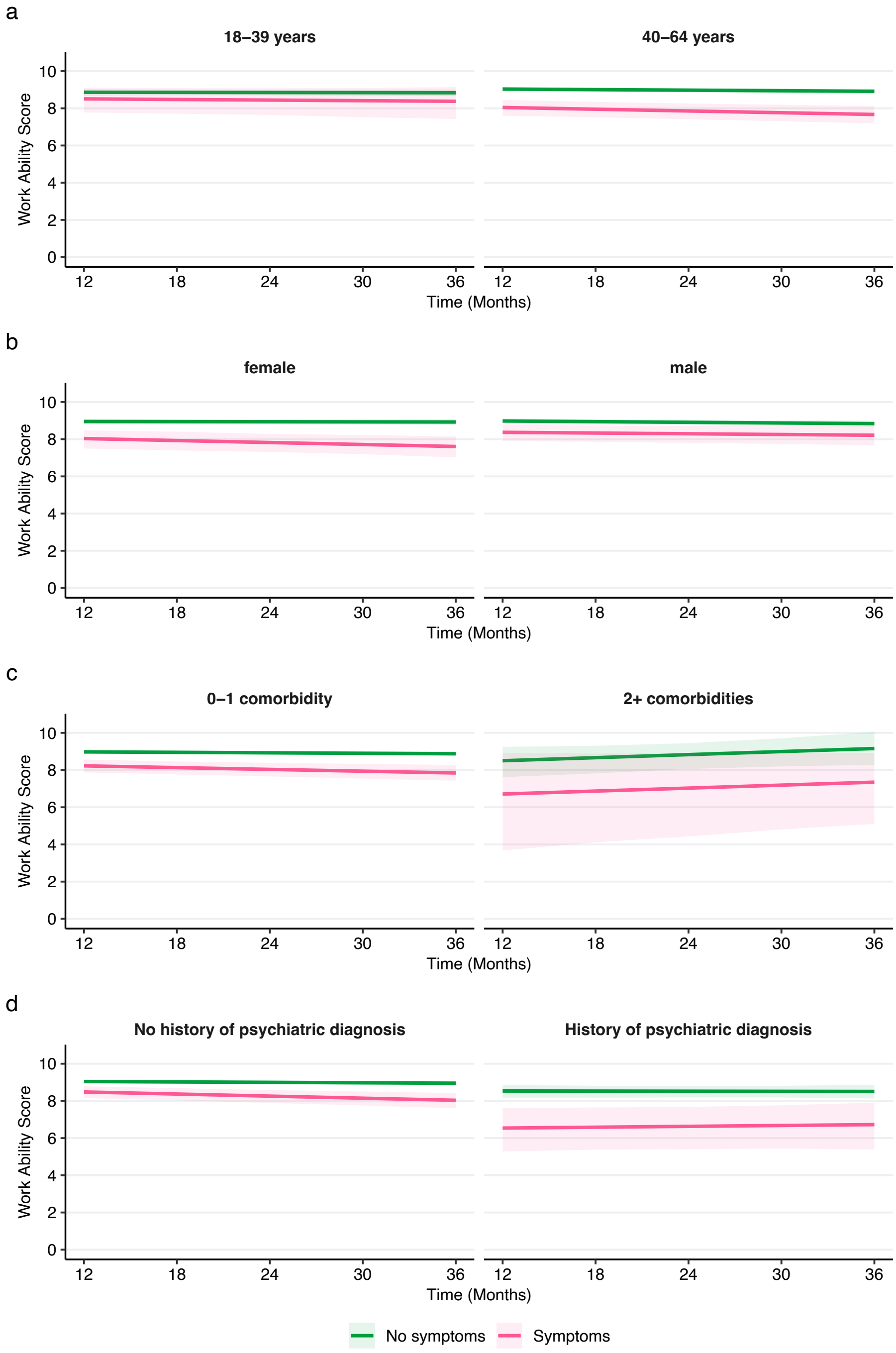

Supplementary Table 9. Categorized work ability scores over time, stratified by presence of self-reported COVID-19 related symptoms and (non-)recovery and health impairment at 12 months after diagnosis of primary infection, in the main and sensitivity analyses.

			IPCW		Available case (unweighted)		Complete case (unweighted)	
**Outcome**		**Timepoint**	**n/N***	**% (95% CI)**	**n/N**	**% (95% CI)**	**n/N**	**% (95% CI)**
**Current work ability (categorical)**	**Symptoms**							
Poor	*No symptoms*	M12		5.0 (3.5 to 7.2)	27/537	5.0 (3.5 to 7.2)	11/299	3.7 (2.1 to 6.5)
		M18		4.0 (2.6 to 6.3)	17/442	3.8 (2.4 to 6.1)	12/299	4.0 (2.3 to 6.9)
		M24		3.5 (2.1 to 5.8)	15/397	3.8 (2.3 to 6.1)	12/299	4.0 (2.3 to 6.9)
		M30		5.0 (3.2 to 7.7)	17/342	5.0 (3.1 to 7.8)	13/299	4.3 (2.6 to 7.3)
		M36		5.1 (3.2 to 8.0)	17/315	5.4 (3.4 to 8.5)	16/299	5.4 (3.3 to 8.5)
Poor	*Symptoms*	M12		16.8 (11.2 to 24.5)	20/119	16.8 (11.2 to 24.5)	12/70	17.1 (10.1 to 27.6)
		M18		22.9 (16.0 to 31.8)	23/105	21.9 (15.1 to 30.7)	15/70	21.4 (13.4 to 32.4)
		M24		24.0 (16.8 to 33.1)	22/98	22.4 (15.3 to 31.7)	16/70	22.9 (14.6 to 34.0)
		M30		30.9 (22.4 to 41)	24/85	28.2 (19.8 to 38.6)	19/70	27.1 (18.1 to 38.5)
		M36		21.0 (13.5 to 31.2)	15/76	19.7 (12.3 to 30.0)	15/70	21.4 (13.4 to 32.4)
Moderate	*No symptoms*	M12		25.5 (22.0 to 29.4)	137/537	25.5 (22.0 to 29.4)	71/299	23.7 (19.3 to 28.9)
		M18		22.4 (18.8 to 26.5)	102/442	23.1 (19.4 to 27.2)	68/299	22.7 (18.4 to 27.8)
		M24		23.3 (19.5 to 27.6)	88/397	22.2 (18.4 to 26.5)	62/299	20.7 (16.5 to 25.7)
		M30		19.0 (15.3 to 23.3)	66/342	19.3 (15.5 to 23.8)	58/299	19.4 (15.3 to 24.3)
		M36		26.9 (22.4 to 31.8)	81/315	25.7 (21.2 to 30.8)	76/299	25.4 (20.8 to 30.6)
Moderate	*Symptoms*	M12		35.3 (27.3 to 44.2)	42/119	35.3 (27.3 to 44.2)	20/70	28.6 (19.3 to 40.1)
		M18		35.4 (26.9 to 44.9)	38/105	36.2 (27.6 to 45.7)	22/70	31.4 (21.8 to 43.0)
		M24		32.7 (24.4 to 42.2)	33/98	33.7 (25.1 to 43.5)	21/70	30.0 (20.5 to 41.5)
		M30		20.4 (13.4 to 29.8)	18/85	21.2 (13.8 to 31.0)	16/70	22.9 (14.6 to 34.0)
		M36		37.5 (27.6 to 48.5)	30/76	39.5 (29.2 to 50.7)	26/70	37.1 (26.8 to 48.9)
Excellent	*No symptoms*	M12		69.5 (65.4 to 73.2)	373/537	69.5 (65.4 to 73.2)	217/299	72.6 (67.3 to 77.3)
		M18		73.6 (69.3 to 77.4)	323/442	73.1 (68.8 to 77.0)	219/299	73.2 (68.0 to 77.9)
		M24		73.2 (68.7 to 77.2)	294/397	74.1 (69.5 to 78.1)	225/299	75.3 (70.1 to 79.8)
		M30		76.0 (71.4 to 80.1)	259/342	75.7 (70.9 to 80.0)	228/299	76.3 (71.1 to 80.7)
		M36		68 (62.9 to 72.8)	217/315	68.9 (63.6 to 73.7)	207/299	69.2 (63.8 to 74.2)
Excellent	*Symptoms*	M12		47.9 (39.1 to 56.8)	57/119	47.9 (39.1 to 56.8)	38/70	54.3 (42.7 to 65.4)
		M18		41.7 (32.7 to 51.2)	44/105	41.9 (32.9 to 51.5)	33/70	47.1 (35.9 to 58.7)
		M24		43.3 (34.2 to 53.0)	43/98	43.9 (34.5 to 53.7)	33/70	47.1 (35.9 to 58.7)
		M30		48.7 (38.7 to 58.8)	43/85	50.6 (40.2 to 61.0)	35/70	50.0 (38.6 to 61.4)
		M36		41.5 (31.3 to 52.5)	31/76	40.8 (30.4 to 52.0)	29/70	41.4 (30.6 to 53.1)
**Current work ability (categorical)**	**(Non-) recovery and health impairment**							
Poor	*Recovered*	M12		4.1 (2.8 to 6.1)	23/555	4.1 (2.8 to 6.1)	9/310	2.9 (1.5 to 5.4)
		M18		3.4 (2.1 to 5.5)	15/456	3.3 (2.0 to 5.4)	9/310	2.9 (1.5 to 5.4)
		M24		3.1 (1.8 to 5.2)	13/410	3.2 (1.9 to 5.3)	10/310	3.2 (1.8 to 5.8)
		M30		5.5 (3.6 to 8.3)	19/354	5.4 (3.5 to 8.2)	14/310	4.5 (2.7 to 7.4)
		M36		4.7 (3.0 to 7.5)	16/330	4.8 (3.0 to 7.7)	15/310	4.8 (3.0 to 7.8)
Poor	*Mild*	M12		2.8 (0.8 to 9.6)	2/72	2.8 (0.8 to 9.6)	1/42	2.4 (0.1 to 12.3)
		M18		11.2 (5.7 to 21.0)	7/65	10.8 (5.3 to 20.6)	5/42	11.9 (5.2 to 25.0)
		M24		7.2 (3.0 to 16.4)	4/60	6.7 (2.6 to 15.9)	4/42	9.5 (3.8 to 22.1)

				IPCW	Available case (unweighted)		Complete case (unweighted)	
Outcome		Timepoint	n/N*	% (95% CI)	n/N	% (95% CI)	n/N	% (95% CI)
		M30		18.2 (9.9 to 30.9)	8/49	16.3 (8.5 to 29.0)	7/42	16.7 (8.3 to 30.6)
		M36		18.1 (9.5 to 31.8)	7/43	16.3 (8.1 to 30.0)	7/42	16.7 (8.3 to 30.6)
Poor	*Moderate*	M12		76.9 (49.7 to 91.8)	10/13	76.9 (49.7 to 91.8)	8/10	80.0 (49.0 to 94.3)
		M18		85.9 (59.2 to 96.3)	10/12	83.3 (55.2 to 95.3)	8/10	80.0 (49.0 to 94.3)
		M24		92.4 (65.6 to 98.7)	10/11	90.9 (62.3 to 99.5)	9/10	90.0 (59.6 to 99.5)
		M30		57.6 (32.3 to 79.5)	7/12	58.3 (32.0 to 80.7)	6/10	60.0 (31.3 to 83.2)
		M36		35.9 (15.6 to 62.9)	4/11	36.4 (15.2 to 64.6)	4/10	40.0 (16.8 to 68.7)
Poor	*Severe*	M12		87.5 (52.9 to 97.8)	7/8	87.5 (52.9 to 99.4)	3/4	75.0 (30.1 to 98.7)
		M18		85.4 (47.7 to 97.4)	6/7	85.7 (48.7 to 99.3)	3/4	75.0 (30.1 to 98.7)
		M24		86.8 (51.5 to 97.6)	6/7	85.7 (48.7 to 99.3)	3/4	75.0 (30.1 to 98.7)
		M30		77.5 (44.7 to 93.6)	5/7	71.4 (35.9 to 91.8)	3/4	75.0 (30.1 to 98.7)
		M36		73.7 (28.0 to 95.3)	3/4	75.0 (30.1 to 98.7)	3/4	75.0 (30.1 to 98.7)
Moderate	*Recovered*	M12		24.7 (21.3 to 28.4)	137/555	24.7 (21.3 to 28.4)	67/310	21.6 (17.4 to 26.5)
		M18		23.0 (19.4 to 27.1)	108/456	23.7 (20.0 to 27.8)	72/310	23.2 (18.9 to 28.2)
		M24		23.9 (20.1 to 28.2)	95/410	23.2 (19.3 to 27.5)	66/310	21.3 (17.1 to 26.2)
		M30		17.8 (14.3 to 22.0)	64/354	18.1 (14.4 to 22.4)	57/310	18.4 (14.5 to 23.1)
		M36		26.7 (22.4 to 31.5)	86/330	26.1 (21.6 to 31.1)	79/310	25.5 (21.0 to 30.6)
Moderate	*Mild*	M12		52.8 (41.4 to 63.9)	38/72	52.8 (41.4 to 63.9)	21/42	50.0 (35.5 to 64.5)
		M18		41.8 (30.7 to 53.8)	28/65	43.1 (31.8 to 55.2)	17/42	40.5 (27.0 to 55.5)
		M24		41.1 (29.8 to 53.5)	24/60	40.0 (28.6 to 52.6)	16/42	38.1 (25.0 to 53.2)
		M30		32.1 (20.9 to 45.8)	16/49	32.7 (21.2 to 46.6)	15/42	35.7 (23.0 to 50.8)
		M36		38.8 (25.9 to 53.4)	17/43	39.5 (26.4 to 54.4)	16/42	38.1 (25.0 to 53.2)
Moderate	*Moderate*	M12		15.4 (4.3 to 42.2)	2/13	15.4 (4.3 to 42.2)	2/10	20.0 (5.7 to 51.0)
		M18		6.9 (1.1 to 32.4)	1/12	8.3 (0.4 to 35.4)	1/10	10.0 (0.5 to 40.4)
		M24		7.6 (1.3 to 34.4)	1/11	9.1 (0.5 to 37.7)	1/10	10.0 (0.5 to 40.4)
		M30		23.3 (8.3 to 50.4)	3/12	25.0 (8.9 to 53.2)	2/10	20.0 (5.7 to 51.0)
		M36		64.1 (37.1 to 84.4)	7/11	63.6 (35.4 to 84.8)	6/10	60.0 (31.3 to 83.2)
Moderate	*Severe*	M12		12.5 (2.2 to 47.1)	1/8	12.5 (0.6 to 47.1)	1/4	25.0 (1.3 to 69.9)
		M18		0.0 (0.0 to 36.2)	NA	NA	NA	NA
		M24		0.0 (0.0 to 33.3)	NA	NA	NA	NA
		M30		11.4 (2.0 to 44.1)	1/7	14.3 (0.7 to 51.3)	NA	NA
		M36		26.3 (4.7 to 72.0)	1/4	25.0 (1.3 to 69.9)	1/4	25.0 (1.3 to 69.9)
Excellent	*Recovered*	M12		71.2 (67.3 to 74.8)	395/555	71.2 (67.3 to 74.8)	234/310	75.5 (70.4 to 79.9)
		M18		73.6 (69.4 to 77.4)	333/456	73.0 (68.8 to 76.9)	229/310	73.9 (68.7 to 78.4)
		M24		73.0 (68.6 to 77.0)	302/410	73.7 (69.2 to 77.7)	234/310	75.5 (70.4 to 79.9)
		M30		76.7 (72.2 to 80.7)	271/354	76.6 (71.9 to 80.7)	239/310	77.1 (72.1 to 81.4)
		M36		68.6 (63.6 to 73.2)	228/330	69.1 (63.9 to 73.8)	216/310	69.7 (64.3 to 74.5)
Excellent	*Mild*	M12		44.4 (33.5 to 55.9)	32/72	44.4 (33.5 to 55.9)	20/42	47.6 (33.4 to 62.3)
		M18		47.0 (35.5 to 58.8)	30/65	46.2 (34.6 to 58.1)	20/42	47.6 (33.4 to 62.3)
		M24		51.7 (39.5 to 63.6)	32/60	53.3 (40.9 to 65.4)	22/42	52.4 (37.7 to 66.6)
		M30		49.7 (36.5 to 63.0)	25/49	51.0 (37.5 to 64.4)	20/42	47.6 (33.4 to 62.3)
		M36		43.1 (29.7 to 57.7)	19/43	44.2 (30.4 to 58.9)	19/42	45.2 (31.2 to 60.1)
Excellent	*Moderate*	M12		7.7 (1.4 to 33.3)	1/13	7.7 (0.4 to 33.3)	NA	NA
		M18		7.2 (1.2 to 32.7)	1/12	8.3 (0.4 to 35.4)	1/10	10.0 (0.5 to 40.4)
		M24		0.0 (0.0 to 24.1)	NA	NA	NA	NA

			IPCW		Available case (unweighted)		Complete case (unweighted)	
**Outcome**		**Timepoint**	**n/N***	**% (95% CI)**	**n/N**	**% (95% CI)**	**n/N**	**% (95% CI)**
		M30		19.1 (6.1 to 46.1)	2/12	16.7 (4.7 to 44.8)	2/10	20.0 (5.7 to 51.0)
		M36		0.0 (0.0 to 23.8)	NA	NA	NA	NA
Excellent	*Severe*	M12		0.0 (0.0 to 32.4)	NA	NA	NA	NA
		M18		14.6 (2.6 to 52.3)	1/7	14.3 (0.7 to 51.3)	1/4	25.0 (1.3 to 69.9)
		M24		13.2 (2.4 to 48.5)	1/7	14.3 (0.7 to 51.3)	1/4	25.0 (1.3 to 69.9)
		M30		11.2 (2.0 to 43.8)	1/7	14.3 (0.7 to 51.3)	1/4	25.0 (1.3 to 69.9)
		M36		0.0 (0.0 to 50.9)	NA	NA	NA	NA

Legend: */NA, Not applicable; IPCW, inverse probability of censoring weighting; n, number; N, total number; CI, confidence interval; M, month

Supplementary Table 10. Work ability related to physical demands over time, stratified by presence of self-reported COVID-19 related symptoms and (non-)recovery and health impairment at 12 months after diagnosis of primary infection, in the main and sensitivity analyses.

			IPCW		Available case (unweighted)		Complete case (unweighted)	
**Outcome**		**Timepoint**	**n/N***	**% (95% CI)**	**n/N**	**% (95% CI)**	**n/N**	**% (95% CI)**
**Work ability related to physical demands**	**Symptoms**							
Very bad	*No symptoms*	M12		0.4 (0.1 to 1.3)	2/537	0.4 (0.1 to 1.3)	1/297	0.3 (0.0 to 1.9)
		M18		0.0 (0.0 to 0.8)	NA	NA	NA	NA
		M24		0.5 (0.1 to 1.7)	2/397	0.5 (0.1 to 1.8)	2/297	0.7 (0.2 to 2.4)
		M30		0.3 (0.0 to 1.5)	1/343	0.3 (0.0 to 1.6)	1/297	0.3 (0.0 to 1.9)
		M36		0.4 (0.1 to 1.8)	1/315	0.3 (0.0 to 1.8)	1/297	0.3 (0.0 to 1.9)
Very bad	*Symptoms*	M12		2.5 (0.9 to 7.2)	3/118	2.5 (0.9 to 7.2)	1/70	1.4 (0.1 to 7.7)
		M18		4.4 (1.8 to 10.2)	4/104	3.8 (1.5 to 9.5)	3/70	4.3 (1.5 to 11.9)
		M24		5.3 (2.3 to 11.5)	4/97	4.1 (1.6 to 10.1)	3/70	4.3 (1.5 to 11.9)
		M30		8.1 (4.1 to 15.6)	6/85	7.1 (3.3 to 14.6)	5/70	7.1 (3.1 to 15.7)
		M36		4.0 (1.4 to 10.7)	3/77	3.9 (1.3 to 10.8)	3/70	4.3 (1.5 to 11.9)
Rather bad	*No symptoms*	M12		1.1 (0.5 to 2.4)	6/537	1.1 (0.5 to 2.4)	3/297	1.0 (0.3 to 2.9)
		M18		0.6 (0.2 to 1.9)	3/439	0.7 (0.2 to 2.0)	2/297	0.7 (0.2 to 2.4)
		M24		0.4 (0.1 to 1.6)	2/397	0.5 (0.1 to 1.8)	2/297	0.7 (0.2 to 2.4)
		M30		1.1 (0.4 to 2.7)	4/343	1.2 (0.5 to 3.0)	2/297	0.7 (0.2 to 2.4)
		M36		0.2 (0.0 to 1.6)	1/315	0.3 (0.0 to 1.8)	1/297	0.3 (0.0 to 1.9)
Rather bad	*Symptoms*	M12		4.2 (1.8 to 9.5)	5/118	4.2 (1.8 to 9.5)	4/70	5.7 (2.2 to 13.8)
		M18		5.1 (2.3 to 11.1)	5/104	4.8 (2.1 to 10.8)	2/70	2.9 (0.8 to 9.8)
		M24		2.9 (1.0 to 8.2)	3/97	3.1 (1.1 to 8.7)	1/70	1.4 (0.1 to 7.7)
		M30		7.3 (3.5 to 14.5)	6/85	7.1 (3.3 to 14.6)	5/70	7.1 (3.1 to 15.7)
		M36		3.5 (1.1 to 10.0)	3/77	3.9 (1.3 to 10.8)	2/70	2.9 (0.8 to 9.8)
Moderate	*No symptoms*	M12		3.9 (2.6 to 5.9)	21/537	3.9 (2.6 to 5.9)	13/297	4.4 (2.6 to 7.3)
		M18		4.8 (3.2 to 7.2)	20/439	4.6 (3.0 to 6.9)	16/297	5.4 (3.3 to 8.6)
		M24		4.8 (3.2 to 7.3)	19/397	4.8 (3.1 to 7.4)	14/297	4.7 (2.8 to 7.8)
		M30		5.4 (3.5 to 8.2)	19/343	5.5 (3.6 to 8.5)	17/297	5.7 (3.6 to 9.0)
		M36		4.4 (2.7 to 7.1)	14/315	4.4 (2.7 to 7.3)	13/297	4.4 (2.6 to 7.3)
Moderate	*Symptoms*	M12		20.3 (14.1 to 28.5)	24/118	20.3 (14.1 to 28.5)	13/70	18.6 (11.2 to 29.2)
		M18		18.7 (12.4 to 27.2)	20/104	19.2 (12.8 to 27.8)	13/70	18.6 (11.2 to 29.2)
		M24		23.9 (16.6 to 33.1)	23/97	23.7 (16.4 to 33.1)	17/70	24.3 (15.8 to 35.5)
		M30		18.7 (12.0 to 27.9)	15/85	17.6 (11.0 to 27.1)	11/70	15.7 (9.0 to 26.0)
		M36		14.1 (8.1 to 23.3)	11/77	14.3 (8.2 to 23.8)	9/70	12.9 (6.9 to 22.7)
Rather good	*No symptoms*	M12		21.4 (18.2 to 25.1)	115/537	21.4 (18.2 to 25.1)	55/297	18.5 (14.5 to 23.3)
		M18		23.2 (19.5 to 27.3)	102/439	23.2 (19.5 to 27.4)	67/297	22.6 (18.2 to 27.6)
		M24		21.4 (17.7 to 25.6)	82/397	20.7 (17.0 to 24.9)	60/297	20.2 (16.0 to 25.1)
		M30		20.1 (16.3 to 24.5)	68/343	19.8 (16.0 to 24.4)	58/297	19.5 (15.4 to 24.4)
		M36		23.2 (19.0 to 28)	72/315	22.9 (18.6 to 27.8)	69/297	23.2 (18.8 to 28.4)
Rather good	*Symptoms*	M12		33.1 (25.2 to 42.0)	39/118	33.1 (25.2 to 42.0)	18/70	25.7 (16.9 to 37.0)
		M18		28.2 (20.4 to 37.4)	29/104	27.9 (20.2 to 37.2)	17/70	24.3 (15.8 to 35.5)
		M24		27.7 (19.9 to 37.1)	28/97	28.9 (20.8 to 38.6)	19/70	27.1 (18.1 to 38.5)

				IPCW	Available case (unweighted)		Complete case (unweighted)	
Outcome		Timepoint	n/N*	% (95% CI)	n/N	% (95% CI)	n/N	% (95% CI)
		M30		15.9 (9.8 to 24.7)	16/85	18.8 (11.9 to 28.4)	13/70	18.6 (11.2 to 29.2)
		M36		40.4 (30.4 to 51.3)	32/77	41.6 (31.2 to 52.7)	30/70	42.9 (31.9 to 54.5)
Very good	*No symptoms*	M12		73.2 (69.3 to 76.8)	393/537	73.2 (69.3 to 76.8)	225/297	75.8 (70.6 to 80.3)
		M18		71.3 (67.0 to 75.3)	314/439	71.5 (67.1 to 75.5)	212/297	71.4 (66.0 to 76.2)
		M24		72.9 (68.4 to 76.9)	292/397	73.6 (69.0 to 77.6)	219/297	73.7 (68.5 to 78.4)
		M30		73.1 (68.4 to 77.4)	251/343	73.2 (68.3 to 77.6)	219/297	73.7 (68.5 to 78.4)
		M36		71.8 (66.8 to 76.3)	227/315	72.1 (66.9 to 76.7)	213/297	71.7 (66.3 to 76.5)
Very good	*Symptoms*	M12		39.8 (31.5 to 48.8)	47/118	39.8 (31.5 to 48.8)	34/70	48.6 (37.2 to 60.0)
		M18		43.7 (34.5 to 53.2)	46/104	44.2 (35.1 to 53.8)	35/70	50.0 (38.6 to 61.4)
		M24		40.3 (31.2 to 50.0)	39/97	40.2 (31.0 to 50.2)	30/70	42.9 (31.9 to 54.5)
		M30		50.0 (40.0 to 60.1)	42/85	49.4 (39.0 to 59.8)	36/70	51.4 (40.0 to 62.8)
		M36		38.1 (28.3 to 49.0)	28/77	36.4 (26.5 to 47.5)	26/70	37.1 (26.8 to 48.9)
**Work ability related to physical demands**	**(Non-) recovery and health impairment**							
Very bad	*Recovered*	M12		0.4 (0.1 to 1.3)	2/555	0.4 (0.1 to 1.3)	1/308	0.3 (0.0 to 1.8)
		M18		0.0 (0.0 to 0.8)	NA	NA	NA	NA
		M24		0.4 (0.1 to 1.6)	2/410	0.5 (0.1 to 1.8)	2/308	0.6 (0.2 to 2.3)
		M30		0.5 (0.1 to 1.9)	2/355	0.6 (0.2 to 2.0)	2/308	0.6 (0.2 to 2.3)
		M36		0.3 (0.1 to 1.7)	1/331	0.3 (0.0 to 1.7)	1/308	0.3 (0.0 to 1.8)
Very bad	*Mild*	M12		0.0 (0.0 to 5.1)	NA	NA	NA	NA
		M18		0.0 (0.0 to 5.4)	NA	NA	NA	NA
		M24		0.0 (0.0 to 5.8)	NA	NA	NA	NA
		M30		1.6 (0.2 to 9.6)	1/50	2.0 (0.1 to 10.5)	1/43	2.3 (0.1 to 12.1)
		M36		0.0 (0.0 to 8.0)	NA	NA	NA	NA
Very bad	*Moderate*	M12		7.7 (1.4 to 33.3)	1/13	7.7 (0.4 to 33.3)	1/10	10.0 (0.5 to 40.4)
		M18		12.0 (2.9 to 38.5)	1/12	8.3 (0.4 to 35.4)	1/10	10.0 (0.5 to 40.4)
		M24		12.1 (2.8 to 39.7)	1/11	9.1 (0.5 to 37.7)	1/10	10.0 (0.5 to 40.4)
		M30		6.2 (0.9 to 31.3)	1/12	8.3 (0.4 to 35.4)	1/10	10.0 (0.5 to 40.4)
		M36		13.2 (3.3 to 40.6)	1/11	9.1 (0.5 to 37.7)	1/10	10.0 (0.5 to 40.4)
Very bad	*Severe*	M12		14.3 (2.6 to 51.3)	1/7	14.3 (0.7 to 51.3)	NA	NA
		M18		52.5 (20.0 to 83.1)	3/6	50.0 (18.8 to 81.2)	2/3	66.7 (20.8 to 98.3)
		M24		50.7 (21.6 to 79.3)	3/7	42.9 (15.8 to 75.0)	2/3	66.7 (20.8 to 98.3)
		M30		63.0 (30.4 to 86.9)	3/6	50.0 (18.8 to 81.2)	2/3	66.7 (20.8 to 98.3)
		M36		42.5 (10.9 to 81.8)	2/4	50.0 (15.0 to 85.0)	2/3	66.7 (20.8 to 98.3)
Rather bad	*Recovered*	M12		0.7 (0.3 to 1.8)	4/555	0.7 (0.3 to 1.8)	2/308	0.6 (0.2 to 2.3)
		M18		0.4 (0.1 to 1.6)	2/453	0.4 (0.1 to 1.6)	1/308	0.3 (0.0 to 1.8)
		M24		0.2 (0.0 to 1.3)	1/410	0.2 (0.0 to 1.4)	1/308	0.3 (0.0 to 1.8)
		M30		1.4 (0.6 to 3.2)	5/355	1.4 (0.6 to 3.3)	3/308	1.0 (0.3 to 2.8)
		M36		0.0 (0.0 to 1.1)	NA	NA	NA	NA
Rather bad	*Mild*	M12		0.0 (0.0 to 5.1)	NA	NA	NA	NA
		M18		1.5 (0.3 to 8.0)	1/65	1.5 (0.1 to 8.2)	1/43	2.3 (0.1 to 12.1)
		M24		0.0 (0.0 to 5.8)	NA	NA	NA	NA
		M30		1.7 (0.3 to 9.9)	1/50	2.0 (0.1 to 10.5)	1/43	2.3 (0.1 to 12.1)
		M36		3.7 (0.9 to 13.9)	2/43	4.7 (1.3 to 15.5)	2/43	4.7 (1.3 to 15.5)
Rather bad	*Moderate*	M12		15.4 (4.3 to 42.2)	2/13	15.4 (4.3 to 42.2)	2/10	20.0 (5.7 to 51.0)

			IPCW		Available case (unweighted)		Complete case (unweighted)	
Outcome		Timepoint	n/N*	% (95% CI)	n/N	% (95% CI)	n/N	% (95% CI)
		M18		16.3 (4.8 to 43.3)	2/12	16.7 (4.7 to 44.8)	NA	NA
		M24		8.7 (1.6 to 35.8)	1/11	9.1 (0.5 to 37.7)	1/10	10.0 (0.5 to 40.4)
		M30		18.2 (5.7 to 45.2)	2/12	16.7 (4.7 to 44.8)	1/10	10.0 (0.5 to 40.4)
		M36		0.0 (0.0 to 23.8)	NA	NA	NA	NA
Rather bad	*Severe*	M12		28.6 (8.2 to 64.1)	2/7	28.6 (8.2 to 64.1)	1/3	33.3 (1.7 to 79.2)
		M18		0.0 (0.0 to 39.9)	NA	NA	NA	NA
		M24		24 (6.5 to 58.8)	2/7	28.6 (8.2 to 64.1)	NA	NA
		M30		0.0 (0.0 to 33.4)	NA	NA	NA	NA
		M36		31.2 (6.3 to 75.1)	1/4	25.0 (1.3 to 69.9)	NA	NA
Moderate	*Recovered*	M12		4.0 (2.6 to 5.9)	22/555	4.0 (2.6 to 5.9)	12/308	3.9 (2.2 to 6.7)
		M18		4.7 (3.1 to 7.1)	21/453	4.6 (3.1 to 7.0)	16/308	5.2 (3.2 to 8.3)
		M24		4.9 (3.2 to 7.4)	20/410	4.9 (3.2 to 7.4)	14/308	4.5 (2.7 to 7.5)
		M30		5.3 (3.5 to 8.0)	19/355	5.4 (3.5 to 8.2)	17/308	5.5 (3.5 to 8.7)
		M36		3.9 (2.4 to 6.5)	13/331	3.9 (2.3 to 6.6)	11/308	3.6 (2.0 to 6.3)
Moderate	*Mild*	M12		18.1 (10.9 to 28.5)	13/72	18.1 (10.9 to 28.5)	9/43	20.9 (11.4 to 35.2)
		M18		15.8 (9.0 to 26.4)	10/65	15.4 (8.6 to 26.1)	7/43	16.3 (8.1 to 30.0)
		M24		19.6 (11.6 to 31.1)	11/60	18.3 (10.6 to 29.9)	10/43	23.3 (13.2 to 37.7)
		M30		18.5 (10.2 to 31.1)	8/50	16.0 (8.3 to 28.5)	7/43	16.3 (8.1 to 30.0)
		M36		16.5 (8.4 to 30.0)	7/43	16.3 (8.1 to 30.0)	7/43	16.3 (8.1 to 30.0)
Moderate	*Moderate*	M12		46.2 (23.2 to 70.9)	6/13	46.2 (23.2 to 70.9)	4/10	40.0 (16.8 to 68.7)
		M18		48.5 (25.0 to 72.8)	6/12	50.0 (25.4 to 74.6)	6/10	60.0 (31.3 to 83.2)
		M24		59.2 (32.7 to 81.2)	7/11	63.6 (35.4 to 84.8)	6/10	60.0 (31.3 to 83.2)
		M30		36.6 (16.5 to 62.8)	4/12	33.3 (13.8 to 60.9)	4/10	40.0 (16.8 to 68.7)
		M36		28.9 (11.3 to 56.6)	4/11	36.4 (15.2 to 64.6)	3/10	30.0 (10.8 to 60.3)
Moderate	*Severe*	M12		42.9 (15.8 to 75)	3/7	42.9 (15.8 to 75.0)	1/3	33.3 (1.7 to 79.2)
		M18		30.3 (8.1 to 68.2)	2/6	33.3 (9.7 to 70.0)	NA	NA
		M24		12.2 (2.1 to 47.4)	1/7	14.3 (0.7 to 51.3)	NA	NA
		M30		24.1 (6.6 to 58.9)	2/6	33.3 (9.7 to 70.0)	NA	NA
		M36		0.0 (0.0 to 50.9)	NA	NA	NA	NA
Rather good	*Recovered*	M12		21.3 (18.1 to 24.9)	118/555	21.3 (18.1 to 24.9)	52/308	16.9 (13.1 to 21.5)
		M18		22.6 (19.0 to 26.6)	102/453	22.5 (18.9 to 26.6)	65/308	21.1 (16.9 to 26.0)
		M24		21.2 (17.6 to 25.3)	85/410	20.7 (17.1 to 24.9)	62/308	20.1 (16.0 to 25.0)
		M30		18.7 (15.1 to 22.9)	66/355	18.6 (14.9 to 23.0)	56/308	18.2 (14.3 to 22.9)
		M36		23.6 (19.5 to 28.3)	78/331	23.6 (19.3 to 28.4)	73/308	23.7 (19.3 to 28.8)
Rather good	*Mild*	M12		41.7 (31.0 to 53.2)	30/72	41.7 (31.0 to 53.2)	17/43	39.5 (26.4 to 54.4)
		M18		38.2 (27.4 to 50.2)	25/65	38.5 (27.6 to 50.6)	15/43	34.9 (22.4 to 49.8)
		M24		34.4 (23.9 to 46.8)	21/60	35.0 (24.2 to 47.6)	14/43	32.6 (20.5 to 47.5)
		M30		26.4 (16.3 to 39.8)	14/50	28.0 (17.5 to 41.7)	12/43	27.9 (16.7 to 42.7)
		M36		43.5 (30.0 to 58.0)	19/43	44.2 (30.4 to 58.9)	19/43	44.2 (30.4 to 58.9)
Rather good	*Moderate*	M12		30.8 (12.7 to 57.6)	4/13	30.8 (12.7 to 57.6)	3/10	30.0 (10.8 to 60.3)
		M18		23.2 (8.2 to 50.4)	3/12	25.0 (8.9 to 53.2)	3/10	30.0 (10.8 to 60.3)
		M24		20.0 (6.3 to 48.2)	2/11	18.2 (5.1 to 47.7)	2/10	20.0 (5.7 to 51.0)
		M30		26.9 (10.3 to 53.9)	4/12	33.3 (13.8 to 60.9)	3/10	30.0 (10.8 to 60.3)
		M36		57.9 (31.8 to 80.1)	6/11	54.5 (28.0 to 78.7)	6/10	60.0 (31.3 to 83.2)
Rather good	*Severe*	M12		14.3 (2.6 to 51.3)	1/7	14.3 (0.7 to 51.3)	1/3	33.3 (1.7 to 79.2)

			IPCW		Available case (unweighted)		Complete case (unweighted)	
Outcome		Timepoint	n/N*	% (95% CI)	n/N	% (95% CI)	n/N	% (95% CI)
		M18		17.2 (3.1 to 57.4)	1/6	16.7 (0.9 to 56.4)	1/3	33.3 (1.7 to 79.2)
		M24		13.2 (2.4 to 48.5)	1/7	14.3 (0.7 to 51.3)	1/3	33.3 (1.7 to 79.2)
		M30		0.0 (0.0 to 33.4)	NA	NA	NA	NA
		M36		26.3 (4.7 to 72.0)	1/4	25.0 (1.3 to 69.9)	1/3	33.3 (1.7 to 79.2)
Very good	*Recovered*	M12		73.7 (69.9 to 77.2)	409/555	73.7 (69.9 to 77.2)	241/308	78.2 (73.3 to 82.5)
		M18		72.3 (68.0 to 76.2)	328/453	72.4 (68.1 to 76.3)	226/308	73.4 (68.2 to 78.0)
		M24		73.2 (68.9 to 77.2)	302/410	73.7 (69.2 to 77.7)	229/308	74.4 (69.2 to 78.9)
		M30		74.1 (69.5 to 78.2)	263/355	74.1 (69.3 to 78.4)	230/308	74.7 (69.5 to 79.2)
		M36		72.1 (67.3 to 76.5)	239/331	72.2 (67.1 to 76.8)	223/308	72.4 (67.2 to 77.1)
Very good	*Mild*	M12		40.3 (29.7 to 51.8)	29/72	40.3 (29.7 to 51.8)	17/43	39.5 (26.4 to 54.4)
		M18		44.6 (33.3 to 56.5)	29/65	44.6 (33.2 to 56.7)	20/43	46.5 (32.5 to 61.1)
		M24		46.0 (34.2 to 58.2)	28/60	46.7 (34.6 to 59.1)	19/43	44.2 (30.4 to 58.9)
		M30		51.8 (38.5 to 64.9)	26/50	52.0 (38.5 to 65.2)	22/43	51.2 (36.8 to 65.4)
		M36		36.3 (23.8 to 51)	15/43	34.9 (22.4 to 49.8)	15/43	34.9 (22.4 to 49.8)
Very good	*Moderate*	M12		0.0 (0.0 to 22.8)	NA	NA	NA	NA
		M18		0.0 (0.0 to 22.9)	NA	NA	NA	NA
		M24		0.0 (0.0 to 24.1)	NA	NA	NA	NA
		M30		12.2 (3 to 38.6)	1/12	8.3 (0.4 to 35.4)	1/10	10.0 (0.5 to 40.4)
		M36		0.0 (0.0 to 23.8)	NA	NA	NA	NA
Very good	*Severe*	M12		0.0 (0.0 to 35.4)	NA	NA	NA	NA
		M18		0.0 (0.0 to 39.9)	NA	NA	NA	NA
		M24		0.0 (0.0 to 33.3)	NA	NA	NA	NA
		M30		12.9 (2.3 to 48.3)	1/6	16.7 (0.9 to 56.4)	1/3	33.3 (1.7 to 79.2)
		M36		0.0 (0.0 to 50.9)	NA	NA	NA	NA

Legend: */NA, Not applicable; IPCW, inverse probability of censoring weighting; n, number; N, total number; CI, confidence interval; M, month

Supplementary Table 11. Work ability related to mental demands over time, stratified by presence of self-reported COVID-19 related symptoms and (non-)recovery and health impairment at 12 months after diagnosis of primary infection, in the main and sensitivity analyses.

				IPCW		Available case (unweighted)		Complete case (unweighted)
**Outcome**		**Timepoint**	**n/N***	**% (95%CI)**	**n/N**	**% (95%CI)**	**n/N**	**% (95%CI)**
**Work ability related to mental demands**	**Symptoms**							
Very bad	*No symptoms*	M12		0.4 (0.1 to 1.4)	2/535	0.4 (0.1 to 1.4)	1/293	0.3 (0.0 to 1.9)
		M18		0.2 (0.0 to 1.2)	1/439	0.2 (0.0 to 1.3)	1/293	0.3 (0.0 to 1.9)
		M24		0.4 (0.1 to 1.7)	2/396	0.5 (0.1 to 1.8)	2/293	0.7 (0.2 to 2.5)
		M30		0.9 (0.3 to 2.5)	3/342	0.9 (0.3 to 2.5)	2/293	0.7 (0.2 to 2.5)
		M36		0.0 (0.0 to 1.1)	NA	NA	NA	NA
Very bad	*Symptoms*	M12		1.7 (0.5 to 6.0)	2/118	1.7 (0.5 to 6.0)	NA	NA
		M18		2.1 (0.6 to 7.0)	2/103	1.9 (0.5 to 6.8)	1/70	1.4 (0.1 to 7.7)
		M24		1.9 (0.5 to 6.8)	2/96	2.1 (0.6 to 7.3)	1/70	1.4 (0.1 to 7.7)
		M30		7.8 (3.9 to 15.2)	5/85	5.9 (2.5 to 13.0)	4/70	5.7 (2.2 to 13.8)
		M36		5.4 (2.2 to 12.7)	4/77	5.2 (2.0 to 12.6)	3/70	4.3 (1.5 to 11.9)
Rather bad	*No symptoms*	M12		0.4 (0.1 to 1.4)	2/535	0.4 (0.1 to 1.4)	1/293	0.3 (0.0 to 1.9)
		M18		0.8 (0.3 to 2.1)	3/439	0.7 (0.2 to 2.0)	3/293	1.0 (0.3 to 3.0)
		M24		0.5 (0.1 to 1.8)	2/396	0.5 (0.1 to 1.8)	2/293	0.7 (0.2 to 2.5)
		M30		0.3 (0.1 to 1.6)	1/342	0.3 (0.0 to 1.6)	1/293	0.3 (0.0 to 1.9)
		M36		1.2 (0.5 to 3.0)	4/315	1.3 (0.5 to 3.2)	4/293	1.4 (0.5 to 3.5)
Rather bad	*Symptoms*	M12		4.2 (1.8 to 9.5)	5/118	4.2 (1.8 to 9.5)	4/70	5.7 (2.2 to 13.8)
		M18		5.1 (2.3 to 11.2)	5/103	4.9 (2.1 to 10.9)	3/70	4.3 (1.5 to 11.9)
		M24		6.1 (2.9 to 12.6)	5/96	5.2 (2.2 to 11.6)	3/70	4.3 (1.5 to 11.9)
		M30		5.6 (2.4 to 12.3)	4/85	4.7 (1.8 to 11.5)	4/70	5.7 (2.2 to 13.8)
		M36		4.7 (1.8 to 11.7)	3/77	3.9 (1.3 to 10.8)	3/70	4.3 (1.5 to 11.9)
Moderate	*No symptoms*	M12		9.2 (7.0 to 11.9)	49/535	9.2 (7.0 to 11.9)	23/293	7.8 (5.3 to 11.5)
		M18		6.9 (4.9 to 9.6)	30/439	6.8 (4.8 to 9.6)	18/293	6.1 (3.9 to 9.5)
		M24		6.6 (4.6 to 9.4)	26/396	6.6 (4.5 to 9.4)	20/293	6.8 (4.5 to 10.3)
		M30		5.2 (3.4 to 8.0)	18/342	5.3 (3.4 to 8.2)	13/293	4.4 (2.6 to 7.4)
		M36		8.4 (5.9 to 11.9)	26/315	8.3 (5.7 to 11.8)	25/293	8.5 (5.8 to 12.3)
Moderate	*Symptoms*	M12		19.5 (13.4 to 27.6)	23/118	19.5 (13.4 to 27.6)	13/70	18.6 (11.2 to 29.2)
		M18		19.6 (13.1 to 28.3)	21/103	20.4 (13.7 to 29.2)	16/70	22.9 (14.6 to 34.0)
		M24		23.6 (16.4 to 32.7)	22/96	22.9 (15.6 to 32.3)	19/70	27.1 (18.1 to 38.5)
		M30		20 (13.1 to 29.3)	17/85	20.0 (12.9 to 29.7)	15/70	21.4 (13.4 to 32.4)
		M36		18.8 (11.8 to 28.7)	14/77	18.2 (11.2 to 28.2)	14/70	20.0 (12.3 to 30.8)
Rather good	*No symptoms*	M12		31.8 (28 to 35.8)	170/535	31.8 (28.0 to 35.8)	89/293	30.4 (25.4 to 35.9)
		M18		32.5 (28.3 to 37.0)	141/439	32.1 (27.9 to 36.6)	103/293	35.2 (29.9 to 40.8)
		M24		29.7 (25.5 to 34.3)	114/396	28.8 (24.5 to 33.4)	81/293	27.6 (22.8 to 33.0)
		M30		27.0 (22.7 to 31.7)	92/342	26.9 (22.5 to 31.8)	82/293	28.0 (23.2 to 33.4)
		M36		30.7 (26.0 to 35.8)	94/315	29.8 (25.1 to 35.1)	88/293	30.0 (25.1 to 35.5)
Rather good	*Symptoms*	M12		39.0 (30.7 to 48.0)	46/118	39.0 (30.7 to 48.0)	25/70	35.7 (25.5 to 47.4)
		M18		38.4 (29.6 to 48.0)	39/103	37.9 (29.1 to 47.5)	25/70	35.7 (25.5 to 47.4)
		M24		30.3 (22.2 to 39.8)	30/96	31.2 (22.9 to 41.1)	23/70	32.9 (23.0 to 44.5)

Outcome		Timepoint	IPCW n/N*	IPCW % (95%CI)	Available case (unweighted) n/N	Available case (unweighted) % (95%CI)	Complete case (unweighted) n/N	Complete case (unweighted) % (95%CI)
		M30		25.5 (17.7 to 35.3)	24/85	28.2 (19.8 to 38.6)	19/70	27.1 (18.1 to 38.5)
		M36		36.7 (27.1 to 47.6)	29/77	37.7 (27.7 to 48.8)	25/70	35.7 (25.5 to 47.4)
Very good	*No symptoms*	M12		58.3 (54.1 to 62.4)	312/535	58.3 (54.1 to 62.4)	179/293	61.1 (55.4 to 66.5)
		M18		59.4 (54.8 to 63.9)	264/439	60.1 (55.5 to 64.6)	168/293	57.3 (51.6 to 62.9)
		M24		63.5 (58.7 to 67.9)	252/396	63.6 (58.8 to 68.2)	188/293	64.2 (58.5 to 69.4)
		M30		66.4 (61.4 to 71.0)	228/342	66.7 (61.5 to 71.5)	195/293	66.6 (61.0 to 71.7)
		M36		60.2 (54.9 to 65.3)	191/315	60.6 (55.1 to 65.9)	176/293	60.1 (54.4 to 65.5)
Very good	*Symptoms*	M12		35.6 (27.5 to 44.6)	42/118	35.6 (27.5 to 44.6)	28/70	40.0 (29.3 to 51.7)
		M18		34.8 (26.3 to 44.4)	36/103	35.0 (26.4 to 44.5)	25/70	35.7 (25.5 to 47.4)
		M24		38.2 (29.3 to 47.9)	37/96	38.5 (29.4 to 48.5)	24/70	34.3 (24.2 to 46.0)
		M30		41.1 (31.6 to 51.3)	35/85	41.2 (31.3 to 51.8)	28/70	40.0 (29.3 to 51.7)
		M36		34.3 (24.9 to 45.2)	27/77	35.1 (25.3 to 46.2)	25/70	35.7 (25.5 to 47.4)
**Work ability related to mental demands**	**(Non-) recovery and health impairment**							
Very bad	*Recovered*	M12		0.4 (0.1 to 1.3)	2/553	0.4 (0.1 to 1.3)	1/305	0.3 (0.0 to 1.8)
		M18		0.2 (0.0 to 1.2)	1/453	0.2 (0.0 to 1.2)	1/305	0.3 (0.0 to 1.8)
		M24		0.4 (0.1 to 1.6)	2/410	0.5 (0.1 to 1.8)	2/305	0.7 (0.2 to 2.4)
		M30		0.8 (0.3 to 2.4)	3/354	0.8 (0.3 to 2.5)	2/305	0.7 (0.2 to 2.4)
		M36		0 (0 to 1.1)	NA	NA	NA	NA
Very bad	*Mild*	M12		0 (0 to 5.1)	NA	NA	NA	NA
		M18		0 (0 to 5.5)	NA	NA	NA	NA
		M24		0 (0 to 5.9)	NA	NA	NA	NA
		M30		3.2 (0.8 to 12.1)	2/50	4.0 (1.1 to 13.5)	2/42	4.8 (1.3 to 15.8)
		M36		1.8 (0.3 to 11)	1/43	2.3 (0.1 to 12.1)	1/42	2.4 (0.1 to 12.3)
Very bad	*Moderate*	M12		0 (0 to 22.8)	NA	NA	NA	NA
		M18		0 (0 to 22.9)	NA	NA	NA	NA
		M24		0 (0 to 24.1)	NA	NA	NA	NA
		M30		11.4 (2.7 to 37.7)	1/12	8.3 (0.4 to 35.4)	1/10	10.0 (0.5 to 40.4)
		M36		13.2 (3.3 to 40.6)	1/11	9.1 (0.5 to 37.7)	1/10	10.0 (0.5 to 40.4)
Very bad	*Severe*	M12		14.3 (2.6 to 51.3)	1/7	14.3 (0.7 to 51.3)	NA	NA
		M18		37.1 (11.3 to 73.2)	2/6	33.3 (9.7 to 70.0)	1/3	33.3 (1.7 to 79.2)
		M24		27.6 (7.7 to 63.6)	2/6	33.3 (9.7 to 70.0)	1/3	33.3 (1.7 to 79.2)
		M30		52.8 (23 to 80.7)	2/6	33.3 (9.7 to 70.0)	1/3	33.3 (1.7 to 79.2)
		M36		53.3 (16 to 87.3)	2/4	50.0 (15.0 to 85.0)	1/3	33.3 (1.7 to 79.2)
Rather bad	*Recovered*	M12		0.2 (0 to 1)	1/553	0.2 (0.0 to 1.0)	1/305	0.3 (0.0 to 1.8)
		M18		1 (0.4 to 2.3)	4/453	0.9 (0.3 to 2.2)	4/305	1.3 (0.5 to 3.3)
		M24		0.4 (0.1 to 1.6)	2/410	0.5 (0.1 to 1.8)	1/305	0.3 (0.0 to 1.8)
		M30		1 (0.4 to 2.6)	3/354	0.8 (0.3 to 2.5)	3/305	1.0 (0.3 to 2.9)
		M36		1.1 (0.4 to 2.8)	4/331	1.2 (0.5 to 3.1)	4/305	1.3 (0.5 to 3.3)
Rather bad	*Mild*	M12		0 (0 to 5.1)	NA	NA	NA	NA
		M18		0 (0 to 5.5)	NA	NA	NA	NA
		M24		2 (0.4 to 9.2)	1/59	1.7 (0.1 to 9.0)	1/42	2.4 (0.1 to 12.3)
		M30		3.1 (0.8 to 12)	1/50	2.0 (0.1 to 10.5)	1/42	2.4 (0.1 to 12.3)
		M36		4.1 (1.1 to 14.4)	1/43	2.3 (0.1 to 12.1)	1/42	2.4 (0.1 to 12.3)
Rather bad	*Moderate*	M12		23.1 (8.2 to 50.3)	3/13	23.1 (8.2 to 50.3)	2/10	20.0 (5.7 to 51.0)

Outcome		Timepoint	IPCW		Available case (unweighted)		Complete case (unweighted)	
			n/N*	% (95%CI)	n/N	% (95%CI)	n/N	% (95%CI)
		M18		16.4 (4.8 to 43.4)	2/12	16.7 (4.7 to 44.8)	1/10	10.0 (0.5 to 40.4)
		M24		15.7 (4.3 to 43.7)	2/11	18.2 (5.1 to 47.7)	2/10	20.0 (5.7 to 51.0)
		M30		0 (0 to 22.8)	NA	NA	NA	NA
		M36		0 (0 to 23.8)	NA	NA	NA	NA
Rather bad	*Severe*	M12		14.3 (2.6 to 51.3)	1/7	14.3 (0.7 to 51.3)	1/3	33.3 (1.7 to 79.2)
		M18		0 (0 to 39.9)	NA	NA	NA	NA
		M24		31.5 (9.5 to 66.8)	1/6	16.7 (0.9 to 56.4)	NA	NA
		M30		0 (0 to 33.4)	NA	NA	NA	NA
		M36		20.3 (3 to 67.9)	1/4	25.0 (1.3 to 69.9)	1/3	33.3 (1.7 to 79.2)
Moderate	*Recovered*	M12		8.1 (6.1 to 10.7)	45/553	8.1 (6.1 to 10.7)	19/305	6.2 (4.0 to 9.5)
		M18		6.4 (4.5 to 9)	29/453	6.4 (4.5 to 9.0)	18/305	5.9 (3.8 to 9.1)
		M24		6.5 (4.6 to 9.3)	26/410	6.3 (4.4 to 9.1)	21/305	6.9 (4.5 to 10.3)
		M30		5.5 (3.6 to 8.3)	19/354	5.4 (3.5 to 8.2)	16/305	5.2 (3.3 to 8.4)
		M36		8.9 (6.3 to 12.3)	28/331	8.5 (5.9 to 12.0)	27/305	8.9 (6.2 to 12.6)
Moderate	*Mild*	M12		20.8 (13.1 to 31.6)	15/72	20.8 (13.1 to 31.6)	9/42	21.4 (11.7 to 35.9)
		M18		18.2 (10.8 to 29.3)	12/64	18.8 (11.1 to 30.0)	9/42	21.4 (11.7 to 35.9)
		M24		13.5 (7.1 to 24.2)	8/59	13.6 (7.0 to 24.5)	8/42	19.0 (10.0 to 33.3)
		M30		14.5 (7.4 to 26.5)	7/50	14.0 (7.0 to 26.2)	6/42	14.3 (6.7 to 27.8)
		M36		19.3 (10.4 to 33.2)	8/43	18.6 (9.7 to 32.6)	8/42	19.0 (10.0 to 33.3)
Moderate	*Moderate*	M12		38.5 (17.7 to 64.5)	5/13	38.5 (17.7 to 64.5)	5/10	50.0 (23.7 to 76.3)
		M18		41 (19.5 to 66.7)	5/12	41.7 (19.3 to 68.0)	5/10	50.0 (23.7 to 76.3)
		M24		77.1 (48.9 to 92.2)	8/11	72.7 (43.4 to 90.3)	8/10	80.0 (49.0 to 94.3)
		M30		38.1 (17.5 to 64.1)	4/12	33.3 (13.8 to 60.9)	4/10	40.0 (16.8 to 68.7)
		M36		23.5 (8.2 to 51.4)	3/11	27.3 (9.7 to 56.6)	3/10	30.0 (10.8 to 60.3)
Moderate	*Severe*	M12		57.1 (25 to 84.2)	4/7	57.1 (25.0 to 84.2)	2/3	66.7 (20.8 to 98.3)
		M18		45.8 (15.9 to 79)	3/6	50.0 (18.8 to 81.2)	1/3	33.3 (1.7 to 79.2)
		M24		26 (7 to 62.3)	2/6	33.3 (9.7 to 70.0)	1/3	33.3 (1.7 to 79.2)
		M30		34.3 (11.6 to 67.5)	3/6	50.0 (18.8 to 81.2)	1/3	33.3 (1.7 to 79.2)
		M36		0 (0 to 50.9)	NA	NA	NA	NA
Rather good	*Recovered*	M12		31.8 (28.1 to 35.8)	176/553	31.8 (28.1 to 35.8)	92/305	30.2 (25.3 to 35.5)
		M18		32.8 (28.6 to 37.2)	146/453	32.2 (28.1 to 36.7)	104/305	34.1 (29.0 to 39.6)
		M24		28.8 (24.8 to 33.3)	119/410	29.0 (24.8 to 33.6)	86/305	28.2 (23.4 to 33.5)
		M30		26.7 (22.5 to 31.3)	95/354	26.8 (22.5 to 31.7)	83/305	27.2 (22.5 to 32.5)
		M36		29.8 (25.3 to 34.8)	98/331	29.6 (24.9 to 34.7)	89/305	29.2 (24.4 to 34.5)
Rather good	*Mild*	M12		45.8 (34.8 to 57.3)	33/72	45.8 (34.8 to 57.3)	19/42	45.2 (31.2 to 60.1)
		M18		42.6 (31.4 to 54.7)	27/64	42.2 (30.9 to 54.4)	19/42	45.2 (31.2 to 60.1)
		M24		42.4 (30.8 to 54.9)	24/59	40.7 (29.1 to 53.4)	17/42	40.5 (27.0 to 55.5)
		M30		32.9 (21.7 to 46.5)	16/50	32.0 (20.8 to 45.8)	15/42	35.7 (23.0 to 50.8)
		M36		38.1 (25.3 to 52.8)	17/43	39.5 (26.4 to 54.4)	17/42	40.5 (27.0 to 55.5)
Rather good	*Moderate*	M12		38.5 (17.7 to 64.5)	5/13	38.5 (17.7 to 64.5)	3/10	30.0 (10.8 to 60.3)
		M18		42.5 (20.6 to 67.9)	5/12	41.7 (19.3 to 68.0)	4/10	40.0 (16.8 to 68.7)
		M24		0 (0 to 24.1)	NA	NA	NA	NA
		M30		25.7 (9.7 to 52.8)	4/12	33.3 (13.8 to 60.9)	3/10	30.0 (10.8 to 60.3)
		M36		63.3 (36.4 to 83.9)	7/11	63.6 (35.4 to 84.8)	6/10	60.0 (31.3 to 83.2)
Rather good	*Severe*	M12		14.3 (2.6 to 51.3)	1/7	14.3 (0.7 to 51.3)	NA	NA

Outcome		Timepoint	IPCW n/N*	IPCW % (95%CI)	Available case (unweighted) n/N	Available case (unweighted) % (95%CI)	Complete case (unweighted) n/N	Complete case (unweighted) % (95%CI)
		M18		17.2 (3.1 to 57.4)	1/6	16.7 (0.9 to 56.4)	1/3	33.3 (1.7 to 79.2)
		M24		14.8 (2.7 to 52.2)	1/6	16.7 (0.9 to 56.4)	1/3	33.3 (1.7 to 79.2)
		M30		0 (0 to 33.4)	NA	NA	NA	NA
		M36		26.3 (4.7 to 72)	1/4	25.0 (1.3 to 69.9)	1/3	33.3 (1.7 to 79.2)
Very good	*Recovered*	M12		59.5 (55.4 to 63.5)	329/553	59.5 (55.4 to 63.5)	192/305	63.0 (57.4 to 68.2)
		M18		59.7 (55.2 to 64.1)	273/453	60.3 (55.7 to 64.7)	178/305	58.4 (52.8 to 63.8)
		M24		63.8 (59.2 to 68.2)	261/410	63.7 (58.9 to 68.2)	195/305	63.9 (58.4 to 69.1)
		M30		66 (61.1 to 70.6)	234/354	66.1 (61.0 to 70.8)	201/305	65.9 (60.4 to 71.0)
		M36		60.2 (55 to 65.1)	201/331	60.7 (55.4 to 65.8)	185/305	60.7 (55.1 to 66.0)
Very good	*Mild*	M12		33.3 (23.5 to 44.8)	24/72	33.3 (23.5 to 44.8)	14/42	33.3 (21.0 to 48.4)
		M18		39.1 (28.3 to 51.2)	25/64	39.1 (28.1 to 51.3)	14/42	33.3 (21.0 to 48.4)
		M24		42.1 (30.6 to 54.6)	26/59	44.1 (32.2 to 56.7)	16/42	38.1 (25.0 to 53.2)
		M30		46.3 (33.4 to 59.7)	24/50	48.0 (34.8 to 61.5)	18/42	42.9 (29.1 to 57.8)
		M36		36.7 (24.1 to 51.4)	16/43	37.2 (24.4 to 52.1)	15/42	35.7 (23.0 to 50.8)
Very good	*Moderate*	M12		0 (0 to 22.8)	NA	NA	NA	NA
		M18		0 (0 to 22.9)	NA	NA	NA	NA
		M24		7.2 (1.2 to 33.9)	1/11	9.1 (0.5 to 37.7)	NA	NA
		M30		24.8 (9.1 to 51.9)	3/12	25.0 (8.9 to 53.2)	2/10	20.0 (5.7 to 51.0)
		M36		0 (0 to 23.8)	NA	NA	NA	NA
Very good	*Severe*	M12		0 (0 to 35.4)	NA	NA	NA	NA
		M18		0 (0 to 39.9)	NA	NA	NA	NA
		M24		0 (0 to 35.9)	NA	NA	NA	NA
		M30		12.9 (2.3 to 48.3)	1/6	16.7 (0.9 to 56.4)	1/3	33.3 (1.7 to 79.2)
		M36		0 (0 to 50.9)	NA	NA	NA	NA

Legend: */NA, Not applicable; IPCW, inverse probability of censoring weighting; n, number; N, total number; CI, confidence interval; M, month

Supplementary Table 12. Estimated future work ability, stratified by presence of self-reported COVID-19 related symptoms and (non-)recovery and health impairment at 12 months after diagnosis of primary infection, in the main and sensitivity analyses.

			IPCW		Available case (unweighted)		Complete case (unweighted)	
**Outcome**		**Timepoint**	**n/N***	**% (95% CI)**	**n/N**	**% (95% CI)**	**n/N**	**% (95% CI)**
**Estimated work ability in future (2 years)**	**Symptoms**							
Unlikely	*No symptoms*	M12		2.2 (1.3 to 3.9)	12/537	2.2 (1.3 to 3.9)	3/300	1.0 (0.3 to 2.9)
		M18		3.1 (1.8 to 5.1)	13/439	3.0 (1.7 to 5.0)	10/300	3.3 (1.8 to 6.0)
		M24		1.3 (0.5 to 2.9)	5/396	1.3 (0.5 to 2.9)	2/300	0.7 (0.2 to 2.4)
		M30		1.2 (0.5 to 2.9)	5/343	1.5 (0.6 to 3.4)	4/300	1.3 (0.5 to 3.4)
		M36		1 (0.3 to 2.7)	3/315	1.0 (0.3 to 2.8)	2/300	0.7 (0.2 to 2.4)
Unlikely	*Symptoms*	M12		5.1 (2.4 to 10.7)	6/117	5.1 (2.4 to 10.7)	1/69	1.4 (0.1 to 7.8)
		M18		4.3 (1.7 to 10.1)	4/102	3.9 (1.5 to 9.7)	3/69	4.3 (1.5 to 12.0)
		M24		7.9 (4.1 to 14.8)	7/98	7.1 (3.5 to 14.0)	3/69	4.3 (1.5 to 12.0)
		M30		11 (6.1 to 19.1)	8/85	9.4 (4.8 to 17.5)	5/69	7.2 (3.1 to 15.9)
		M36		12.2 (6.7 to 21.2)	8/75	10.7 (5.5 to 19.7)	7/69	10.1 (5.0 to 19.5)
Not certain	*No symptoms*	M12		4.5 (3 to 6.6)	24/537	4.5 (3.0 to 6.6)	10/300	3.3 (1.8 to 6.0)
		M18		2.2 (1.2 to 4)	10/439	2.3 (1.2 to 4.1)	6/300	2.0 (0.9 to 4.3)
		M24		2.4 (1.3 to 4.4)	11/396	2.8 (1.6 to 4.9)	10/300	3.3 (1.8 to 6.0)
		M30		3.2 (1.8 to 5.5)	11/343	3.2 (1.8 to 5.7)	10/300	3.3 (1.8 to 6.0)
		M36		3.5 (2 to 6)	11/315	3.5 (2.0 to 6.1)	11/300	3.7 (2.1 to 6.4)
Not certain	*Symptoms*	M12		11.1 (6.6 to 18.1)	13/117	11.1 (6.6 to 18.1)	7/69	10.1 (5.0 to 19.5)
		M18		13.2 (8 to 21.1)	13/102	12.7 (7.6 to 20.6)	7/69	10.1 (5.0 to 19.5)
		M24		13.8 (8.4 to 21.8)	13/98	13.3 (7.9 to 21.4)	7/69	10.1 (5.0 to 19.5)
		M30		18.1 (11.6 to 27.3)	15/85	17.6 (11.0 to 27.1)	11/69	15.9 (9.1 to 26.3)
		M36		11.2 (6 to 20.1)	8/75	10.7 (5.5 to 19.7)	7/69	10.1 (5.0 to 19.5)
Relatively certain	*No symptoms*	M12		93.3 (90.9 to 95.1)	501/537	93.3 (90.9 to 95.1)	287/300	95.7 (92.7 to 97.5)
		M18		94.7 (92.3 to 96.4)	416/439	94.8 (92.3 to 96.5)	284/300	94.7 (91.5 to 96.7)
		M24		96.3 (94 to 97.7)	380/396	96.0 (93.5 to 97.5)	288/300	96.0 (93.1 to 97.7)
		M30		95.6 (93 to 97.3)	327/343	95.3 (92.6 to 97.1)	286/300	95.3 (92.3 to 97.2)
		M36		95.6 (92.8 to 97.3)	301/315	95.6 (92.7 to 97.3)	287/300	95.7 (92.7 to 97.5)
Relatively certain	*Symptoms*	M12		83.8 (76 to 89.4)	98/117	83.8 (76.0 to 89.4)	61/69	88.4 (78.8 to 94.0)
		M18		82.5 (74 to 88.7)	85/102	83.3 (74.9 to 89.3)	59/69	85.5 (75.3 to 91.9)
		M24		78.2 (69.3 to 85.1)	78/98	79.6 (70.6 to 86.4)	59/69	85.5 (75.3 to 91.9)
		M30		70.9 (60.8 to 79.2)	62/85	72.9 (62.7 to 81.2)	53/69	76.8 (65.6 to 85.2)
		M36		76.6 (66.1 to 84.6)	59/75	78.7 (68.1 to 86.4)	55/69	79.7 (68.8 to 87.5)
**Estimated work ability in future (2 years)**	**(Non-) recovery and health impairment**							
Unlikely	*Recovered*	M12		2 (1.1 to 3.5)	11/554	2.0 (1.1 to 3.5)	3/314	1.0 (0.3 to 2.8)
		M18		2.6 (1.5 to 4.5)	12/454	2.6 (1.5 to 4.6)	9/314	2.9 (1.5 to 5.4)
		M24		0.9 (0.4 to 2.4)	4/410	1.0 (0.4 to 2.5)	2/314	0.6 (0.2 to 2.3)
		M30		1.2 (0.5 to 2.9)	5/356	1.4 (0.6 to 3.2)	5/314	1.6 (0.7 to 3.7)
		M36		1.3 (0.6 to 3.1)	4/332	1.2 (0.5 to 3.1)	3/314	1.0 (0.3 to 2.8)
Unlikely	*Mild*	M12		4.2 (1.4 to 11.5)	3/72	4.2 (1.4 to 11.5)	NA	NA

			IPCW		Available case (unweighted)		Complete case (unweighted)	
Outcome		Timepoint	n/N*	% (95% CI)	n/N	% (95% CI)	n/N	% (95% CI)
		M18		0 (0 to 5.6)	NA	NA	NA	NA
		M24		1.8 (0.4 to 8.9)	1/60	1.7 (0.1 to 8.9)	NA	NA
		M30		8.3 (3.3 to 19.1)	4/49	8.2 (3.2 to 19.2)	1/40	2.5 (0.1 to 12.9)
		M36		9.2 (3.5 to 21.8)	3/40	7.5 (2.6 to 19.9)	3/40	7.5 (2.6 to 19.9)
Unlikely	*Moderate*	M12		0 (0 to 24.2)	NA	NA	NA	NA
		M18		12.7 (3.2 to 39.3)	1/12	8.3 (0.4 to 35.4)	1/9	11.1 (0.6 to 43.5)
		M24		12.4 (2.9 to 40)	1/11	9.1 (0.5 to 37.7)	1/9	11.1 (0.6 to 43.5)
		M30		0 (0 to 22.8)	NA	NA	NA	NA
		M36		13.2 (3.3 to 40.6)	1/11	9.1 (0.5 to 37.7)	1/9	11.1 (0.6 to 43.5)
Unlikely	*Severe*	M12		37.5 (13.7 to 69.4)	3/8	37.5 (13.7 to 69.4)	1/3	33.3 (1.7 to 79.2)
		M18		52.5 (20 to 83.1)	3/6	50.0 (18.8 to 81.2)	2/3	66.7 (20.8 to 98.3)
		M24		75.7 (41 to 93.3)	5/7	71.4 (35.9 to 91.8)	2/3	66.7 (20.8 to 98.3)
		M30		63 (30.4 to 86.9)	3/6	50.0 (18.8 to 81.2)	2/3	66.7 (20.8 to 98.3)
		M36		73.7 (28 to 95.3)	3/4	75.0 (30.1 to 98.7)	2/3	66.7 (20.8 to 98.3)
Not certain	*Recovered*	M12		4.2 (2.8 to 6.2)	23/554	4.2 (2.8 to 6.2)	9/314	2.9 (1.5 to 5.4)
		M18		2 (1 to 3.7)	9/454	2.0 (1.0 to 3.7)	5/314	1.6 (0.7 to 3.7)
		M24		2.7 (1.5 to 4.7)	12/410	2.9 (1.7 to 5.0)	10/314	3.2 (1.7 to 5.8)
		M30		3.7 (2.2 to 6.1)	13/356	3.7 (2.1 to 6.1)	12/314	3.8 (2.2 to 6.6)
		M36		3.6 (2.1 to 6.1)	11/332	3.3 (1.9 to 5.8)	11/314	3.5 (2.0 to 6.2)
Not certain	*Mild*	M12		5.6 (2.2 to 13.4)	4/72	5.6 (2.2 to 13.4)	2/40	5.0 (1.4 to 16.5)
		M18		6.3 (2.5 to 15)	4/63	6.3 (2.5 to 15.2)	3/40	7.5 (2.6 to 19.9)
		M24		10.6 (5.1 to 20.6)	6/60	10.0 (4.7 to 20.1)	3/40	7.5 (2.6 to 19.9)
		M30		9.2 (3.9 to 20.3)	4/49	8.2 (3.2 to 19.2)	4/40	10.0 (4.0 to 23.1)
		M36		11.2 (4.7 to 24.3)	5/40	12.5 (5.5 to 26.1)	5/40	12.5 (5.5 to 26.1)
Not certain	*Moderate*	M12		41.7 (19.3 to 68)	5/12	41.7 (19.3 to 68.0)	4/9	44.4 (18.9 to 73.3)
		M18		51.8 (27.5 to 75.2)	6/12	50.0 (25.4 to 74.6)	4/9	44.4 (18.9 to 73.3)
		M24		26.7 (9.9 to 54.7)	3/11	27.3 (9.7 to 56.6)	2/9	22.2 (6.3 to 54.7)
		M30		50.8 (26.8 to 74.5)	6/12	50.0 (25.4 to 74.6)	4/9	44.4 (18.9 to 73.3)
		M36		8.1 (1.4 to 34.7)	1/11	9.1 (0.5 to 37.7)	NA	NA
Not certain	*Severe*	M12		25 (7.1 to 59.1)	2/8	25.0 (7.1 to 59.1)	1/3	33.3 (1.7 to 79.2)
		M18		30.3 (8.1 to 68.2)	2/6	33.3 (9.7 to 70.0)	NA	NA
		M24		11.1 (1.8 to 46.3)	1/7	14.3 (0.7 to 51.3)	NA	NA
		M30		24.1 (6.6 to 58.9)	2/6	33.3 (9.7 to 70.0)	NA	NA
		M36		0 (0 to 50.9)	NA	NA	NA	NA
Relatively certain	*Recovered*	M12		93.9 (91.5 to 95.6)	520/554	93.9 (91.5 to 95.6)	302/314	96.2 (93.4 to 97.8)
		M18		95.4 (93.1 to 97)	433/454	95.4 (93.0 to 97.0)	300/314	95.5 (92.7 to 97.3)
		M24		96.4 (94.2 to 97.8)	394/410	96.1 (93.8 to 97.6)	302/314	96.2 (93.4 to 97.8)
		M30		95.1 (92.5 to 96.9)	338/356	94.9 (92.1 to 96.8)	297/314	94.6 (91.5 to 96.6)
		M36		95 (92.3 to 96.9)	317/332	95.5 (92.7 to 97.2)	300/314	95.5 (92.7 to 97.3)
Relatively certain	*Mild*	M12		90.3 (81.3 to 95.2)	65/72	90.3 (81.3 to 95.2)	38/40	95.0 (83.5 to 98.6)
		M18		93.7 (85 to 97.5)	59/63	93.7 (84.8 to 97.5)	37/40	92.5 (80.1 to 97.4)
		M24		87.6 (77.2 to 93.7)	53/60	88.3 (77.8 to 94.2)	37/40	92.5 (80.1 to 97.4)
		M30		82.5 (69.9 to 90.6)	41/49	83.7 (71.0 to 91.5)	35/40	87.5 (73.9 to 94.5)
		M36		79.6 (65 to 89.2)	32/40	80.0 (65.2 to 89.5)	32/40	80.0 (65.2 to 89.5)
Relatively certain	*Moderate*	M12		58.3 (32 to 80.7)	7/12	58.3 (32.0 to 80.7)	5/9	55.6 (26.7 to 81.1)

				IPCW		Available case (unweighted)		Complete case (unweighted)
**Outcome**		**Timepoint**	**n/N***	**% (95% CI)**	**n/N**	**% (95% CI)**	**n/N**	**% (95% CI)**
		M18		35.6 (15.7 to 62)	5/12	41.7 (19.3 to 68.0)	4/9	44.4 (18.9 to 73.3)
		M24		60.9 (34.2 to 82.4)	7/11	63.6 (35.4 to 84.8)	6/9	66.7 (35.4 to 87.9)
		M30		49.2 (25.5 to 73.2)	6/12	50.0 (25.4 to 74.6)	5/9	55.6 (26.7 to 81.1)
		M36		78.7 (50.7 to 93)	9/11	81.8 (52.3 to 94.9)	8/9	88.9 (56.5 to 99.4)
Relatively certain	*Severe*	M12		37.5 (13.7 to 69.4)	3/8	37.5 (13.7 to 69.4)	1/3	33.3 (1.7 to 79.2)
		M18		17.2 (3.1 to 57.4)	1/6	16.7 (0.9 to 56.4)	1/3	33.3 (1.7 to 79.2)
		M24		13.2 (2.4 to 48.5)	1/7	14.3 (0.7 to 51.3)	1/3	33.3 (1.7 to 79.2)
		M30		12.9 (2.3 to 48.3)	1/6	16.7 (0.9 to 56.4)	1/3	33.3 (1.7 to 79.2)
		M36		26.3 (4.7 to 72)	1/4	25.0 (1.3 to 69.9)	1/3	33.3 (1.7 to 79.2)

Legend: */NA, Not applicable; IPCW, inverse probability of censoring weighting; n, number; N, total number; CI, confidence interval; M, month

Supplementary Table 13. Comparison of characteristics of participants completing the month 36 questionnaire versus those that did not.

	Completed M36 (N=416)	Overall (N=667)
**Age**		
Median (IQR)	46.0 (34.0 to 54.0)	43.0 (31.0 to 53.0)
**Age group**		
18-39 years	144 (34.6%)	281 (42.1%)
40-64 years	272 (65.4%)	386 (57.9%)
**Sex**		
Female	225 (54.1%)	362 (54.3%)
Male	191 (45.9%)	305 (45.7%)
**Symptom count at infection**		
Asymptomatic	49 (11.8%)	78 (11.7%)
1-5 symptoms	169 (40.6%)	262 (39.3%)
≥6 symptoms	198 (47.6%)	327 (49.0%)
**Hospitalisation at infection**		
No	410 (98.6%)	658 (98.7%)
Yes	6 (1.4%)	9 (1.3%)
**Smoking status**		
Non-smoker	272 (65.5%)	411 (61.8%)
Ex-smoker	97 (23.4%)	154 (23.2%)
Smoker	46 (11.1%)	100 (15.0%)
Missing	*1*	*2*
**BMI (kg/sqm)**		
Median (IQR)	23.7 (21.5 to 26.3)	23.7 (21.5 to 26.2)
Missing	*4*	*6*
**Comorbidity**		
None	322 (77.4%)	528 (79.2%)
1 comorbidity	80 (19.2%)	113 (16.9%)
2+ comorbidities	14 (3.4%)	26 (3.9%)
**History of psychiatric diagnosis**		
No	350 (85.2%)	565 (86.8%)
Yes	61 (14.8%)	86 (13.2%)
Missing	*5*	*16*
**Education level**		
None or mandatory school	11 (2.7%)	22 (3.3%)
Vocational training or specialized baccalaureate	155 (37.3%)	247 (37.1%)
Higher technical school or college	116 (28.0%)	193 (29.0%)
University	133 (32.0%)	203 (30.5%)
Missing	*1*	*2*
**Employment at infection**		
Employed or self-employed	369 (88.7%)	583 (87.4%)
Student	25 (6.0%)	46 (6.9%)
Housewife/family manager	7 (1.7%)	10 (1.5%)
Unemployed	9 (2.2%)	19 (2.8%)
Disability insurance benefits	4 (1.0%)	4 (0.6%)
Other	2 (0.5%)	5 (0.7%)
**Monthly household income**		
<6'000 CHF	107 (26.5%)	188 (29.2%)
6'000 - 12'000 CHF	183 (45.3%)	281 (43.6%)
>12'000 CHF	114 (28.2%)	175 (27.2%)
Missing	*12*	*23*
**Nationality**		
Swiss	360 (86.5%)	559 (83.8%)
Non-Swiss	56 (13.5%)	108 (16.2%)
**COVID-19 related symptoms at 12 months**		
No symptoms	332 (79.8%)	547 (82.0%)
Symptoms	84 (20.2%)	120 (18.0%)
**(Non-)recovery and health impairment at 12 months**		
Recovered	348 (84.5%)	562 (85.8%)
Mild	46 (11.2%)	72 (11.0%)
Moderate	11 (2.7%)	13 (2.0%)
Severe	7 (1.7%)	8 (1.2%)
Missing	*4*	*12*

Legend: IQR, interquartile range; SD, standard deviation; CHF, Swiss Francs

Supplementary Table 14. Changes in physical and mental performance at work stratified by age, sex, comorbidity count, history of psychiatric diagnoses and presence of COVID-19 related symptoms at 12 months.

	**Overall**	**Age**		**Sex**		**Comorbidity count**		**History of psychiatric diagnoses**		**Presence of COVID-19 related symptoms at 12 months**	
	**(N=416)**	**18-39 years (N=144)**	**40-64 years (N=272)**	**Female (N=225)**	**Male (N=191)**	**0-1 comorbidity (N=402)**	**2+ comorbidities (N=14)**	**No (N=350)**	**Yes (N=61)**	**No symptoms (N=332)**	**Symptoms (N=84)**
**Change in physical performance at work**											
Much worse	7 (1.8%)	2 (1.4%)	5 (2.0%)	5 (2.4%)	2 (1.1%)	7 (1.9%)	0 (0.0%)	4 (1.2%)	3 (5.2%)	2 (0.6%)	5 (6.5%)
Somewhat worse	55 (14.1%)	11 (7.7%)	44 (17.7%)	31 (15.1%)	24 (13.0%)	49 (13.0%)	6 (50.0%)	44 (13.4%)	11 (19.0%)	31 (9.9%)	24 (31.2%)
No change	298 (76.4%)	121 (85.2%)	177 (71.4%)	157 (76.6%)	141 (76.2%)	292 (77.2%)	6 (50.0%)	253 (77.1%)	41 (70.7%)	256 (81.8%)	42 (54.5%)
Somewhat better	13 (3.3%)	4 (2.8%)	9 (3.6%)	5 (2.4%)	8 (4.3%)	13 (3.4%)	0 (0.0%)	11 (3.4%)	2 (3.4%)	9 (2.9%)	4 (5.2%)
Much better	17 (4.4%)	4 (2.8%)	13 (5.2%)	7 (3.4%)	10 (5.4%)	17 (4.5%)	0 (0.0%)	16 (4.9%)	1 (1.7%)	15 (4.8%)	2 (2.6%)
Missing	*26*	*2*	*24*	*20*	*6*	*24*	*2*	*22*	*3*	*19*	*7*
**Self-attribution of worsening to infection**											
No worsening	328 (84.1%)	129 (90.8%)	199 (80.2%)	169 (82.4%)	159 (85.9%)	322 (85.2%)	6 (50.0%)	280 (85.4%)	44 (75.9%)	280 (89.5%)	48 (62.3%)
Yes	18 (4.6%)	3 (2.1%)	15 (6.0%)	14 (6.8%)	4 (2.2%)	16 (4.2%)	2 (16.7%)	12 (3.7%)	6 (10.3%)	7 (2.2%)	11 (14.3%)
No	12 (3.1%)	3 (2.1%)	9 (3.6%)	5 (2.4%)	7 (3.8%)	10 (2.6%)	2 (16.7%)	8 (2.4%)	4 (6.9%)	10 (3.2%)	2 (2.6%)
Does not know	32 (8.2%)	7 (4.9%)	25 (10.1%)	17 (8.3%)	15 (8.1%)	30 (7.9%)	2 (16.7%)	28 (8.5%)	4 (6.9%)	16 (5.1%)	16 (20.8%)
Missing	*26*	*2*	*24*	*20*	*6*	*24*	*2*	*22*	*3*	*19*	*7*
**Change in mental performance at work**											
Much worse	12 (3.1%)	6 (4.2%)	6 (2.5%)	9 (4.5%)	3 (1.6%)	12 (3.2%)	0 (0.0%)	7 (2.2%)	5 (8.8%)	4 (1.3%)	8 (10.8%)
Somewhat worse	47 (12.2%)	15 (10.5%)	32 (13.2%)	31 (15.3%)	16 (8.7%)	45 (12.1%)	2 (16.7%)	36 (11.1%)	11 (19.3%)	30 (9.6%)	17 (23.0%)
No change	304 (79.0%)	112 (78.3%)	192 (79.3%)	149 (73.8%)	155 (84.7%)	294 (78.8%)	10 (83.3%)	264 (81.2%)	37 (64.9%)	261 (83.9%)	43 (58.1%)
Somewhat better	12 (3.1%)	7 (4.9%)	5 (2.1%)	9 (4.5%)	3 (1.6%)	12 (3.2%)	0 (0.0%)	8 (2.5%)	4 (7.0%)	6 (1.9%)	6 (8.1%)
Much better	10 (2.6%)	3 (2.1%)	7 (2.9%)	4 (2.0%)	6 (3.3%)	10 (2.7%)	0 (0.0%)	10 (3.1%)	0 (0.0%)	10 (3.2%)	0 (0.0%)
Missing	*31*	*1*	*30*	*23*	*8*	*29*	*2*	*25*	*4*	*21*	*10*
**Self-attribution of change to infection**											
No worsening	326 (85.1%)	122 (85.3%)	204 (85.0%)	162 (81.0%)	164 (89.6%)	316 (85.2%)	10 (83.3%)	282 (87.0%)	41 (73.2%)	277 (89.4%)	49 (67.1%)
Yes	14 (3.7%)	3 (2.1%)	11 (4.6%)	9 (4.5%)	5 (2.7%)	14 (3.8%)	0 (0.0%)	10 (3.1%)	4 (7.1%)	6 (1.9%)	8 (11.0%)
No	17 (4.4%)	8 (5.6%)	9 (3.8%)	11 (5.5%)	6 (3.3%)	17 (4.6%)	0 (0.0%)	11 (3.4%)	6 (10.7%)	10 (3.2%)	7 (9.6%)
Does not know	26 (6.8%)	10 (7.0%)	16 (6.7%)	18 (9.0%)	8 (4.4%)	24 (6.5%)	2 (16.7%)	21 (6.5%)	5 (8.9%)	17 (5.5%)	9 (12.3%)
Missing	*33*	*1*	*32*	*25*	*8*	*31*	*2*	*26*	*5*	*22*	*11*

Supplementary Table 15. Characteristics of participants reporting ever having a sick leave related to COVID-19 versus those who never had a sick leave.

	Overall (N=405)*	Ever had sick leave (N=284)	Never had sick leave (N=121)
**Age**			
Median (IQR)	46.0 (34.0 to 54.0)	46.5 (34.0 to 54.0)	46.0 (32.0 to 54.0)
**Age group**			
18-39 years	140 (34.6%)	94 (33.1%)	46 (38.0%)
40-64 years	265 (65.4%)	190 (66.9%)	75 (62.0%)
**Sex**			
Female	216 (53.3%)	158 (55.6%)	58 (47.9%)
Male	189 (46.7%)	126 (44.4%)	63 (52.1%)
**Symptom count at infection**			
Asymptomatic	48 (11.9%)	30 (10.6%)	18 (14.9%)
1-5 symptoms	165 (40.7%)	110 (38.7%)	55 (45.5%)
≥6 symptoms	192 (47.4%)	144 (50.7%)	48 (39.7%)
**Hospitalisation at infection**			
Hospitalized	5 (1.2%)	4 (1.4%)	1 (0.8%)
Non-hospitalized	400 (98.8%)	280 (98.6%)	120 (99.2%)
**Comorbidity**			
None	314 (77.5%)	220 (77.5%)	94 (77.7%)
1 comorbidity	78 (19.3%)	55 (19.4%)	23 (19.0%)
2+ comorbidities	13 (3.2%)	9 (3.2%)	4 (3.3%)
**History of psychiatric diagnosis**			
No	344 (85.8%)	235 (83.6%)	109 (90.8%)
Yes	57 (14.2%)	46 (16.4%)	11 (9.2%)
Missing	*4 (1.0%)*	*3 (1.1%)*	*1 (0.8%)*
**Education level**			
None or mandatory school	10 (2.5%)	7 (2.5%)	3 (2.5%)
Vocational training or specialized baccalaureate	149 (36.9%)	103 (36.4%)	46 (38.0%)
Higher technical school or college	115 (28.5%)	87 (30.7%)	28 (23.1%)
University	130 (32.2%)	86 (30.4%)	44 (36.4%)
Missing	*1 (0.2%)*	*1 (0.4%)*	*0 (0%)*
**Employment at infection**			
Employed or self-employed	360 (88.9%)	261 (91.9%)	99 (81.8%)
Student	24 (5.9%)	13 (4.6%)	11 (9.1%)
Housewife/family manager	7 (1.7%)	4 (1.4%)	3 (2.5%)
Unemployed	8 (2.0%)	5 (1.8%)	3 (2.5%)
Disability insurance benefits	4 (1.0%)	1 (0.4%)	3 (2.5%)
Other	2 (0.5%)	0 (0.0%)	2 (1.7%)
**Monthly household income**			
<6'000 CHF	104 (26.5%)	73 (26.4%)	31 (26.5%)
6'000 - 12'000 CHF	177 (45.0%)	125 (45.3%)	52 (44.4%)
>12'000 CHF	112 (28.5%)	78 (28.3%)	34 (29.1%)
Missing	*12 (3.0%)*	*8 (2.8%)*	*4 (3.3%)*
**COVID-19 related symptoms at 12 months**			
No symptoms	327 (80.7%)	223 (78.5%)	104 (86.0%)
Symptoms	78 (19.3%)	61 (21.5%)	17 (14.0%)

* Data on COVID-19 related sick leave was missing from 11 participants.
Legend: IQR, interquartile range; SD, standard deviation; CHF, Swiss Francs

Supplementary Table 16. Results from multivariable regression analysis evaluating risk factors associated with ever having sick leave related to COVID-19 (N= 397).

	OR	95% CI	p-value
**Age at infection (per year increase)**	1.00	0.98 to 1.03	0.76
**Sex**			
Female	Ref.		
Male	0.84	0.54 to 1.31	0.45
**Comorbidity count**			
0-1 comorbidity	Ref.		
2+ comorbidities	0.61	0.17 to 2.44	0.45
**History of psychiatric diagnosis**			
No	Ref.		
Yes	1.81	0.91 to 3.88	0.1
**Baseline EuroQoL visual analog scale (per 1 point increase)**	0.99	0.97 to 1.01	0.37
**Symptom count at infection**			
Asymptomatic	Ref.		
1-5 symptoms	1.36	0.68 to 2.68	0.38
≥6 symptoms	1.81	0.9 to 3.6	0.09
**Education level**			
None or mandatory school	Ref.		
Vocational training or specialized baccalaureate	0.79	0.16 to 3.13	0.75
Higher technical school or college	1.2	0.24 to 4.84	0.81
University	0.77	0.15 to 3.1	0.73
**Self-reported COVID-19 related symptoms**			
No	Ref.		
Yes	1.33	0.72 to 2.52	0.37

Legend: N: number; OR, odds ratio; CI, confidence interval; Ref, reference level

Supplementary Table 17. Results from multivariable regression analysis evaluating risk factors associated with ever having sick leave related to COVID-19 among those who were employed or self-employed at baseline (N= 352).

	OR	95% CI	p-value
**Age at infection (per year increase)**	1.00	0.98 to 1.02	0.93
**Sex**			
Female	Ref.		
Male	0.72	0.44 to 1.18	0.19
**Comorbidity count**			
0-1 comorbidity	Ref.		
2+ comorbidities	0.76	0.19 to 3.86	0.71
**History of psychiatric diagnosis**			
No	Ref.		
Yes	2.53	1.12 to 6.51	0.04
**Baseline EuroQoL visual analog scale (per 1 point increase)**	0.99	0.97 to 1.02	0.62
**Symptom count at infection**			
Asymptomatic	Ref.		
1-5 symptoms	1.51	0.73 to 3.08	0.26
≥6 symptoms	2.17	1.03 to 4.53	0.04
**Education level**			
None or mandatory school	Ref.		
Vocational training or specialized baccalaureate	0.85	0.11 to 4.67	0.86
Higher technical school or college	1.15	0.15 to 6.31	0.88
University	0.66	0.09 to 3.60	0.65
**Self-reported COVID-19 related symptoms**			
No	Ref.		
Yes	1.38	0.70 to 2.85	0.37

Legend: N: number; OR, odds ratio; CI, confidence interval; Ref, reference level
